# *SOX2* Is an Oncogene Activated by Recurrent 3q26.3 Amplifications in Human Lung Squamous Cell Carcinomas

**DOI:** 10.1371/journal.pone.0008960

**Published:** 2010-01-29

**Authors:** Thomas Hussenet, Soraya Dali, Julien Exinger, Ben Monga, Bernard Jost, Doulaye Dembelé, Nadine Martinet, Christelle Thibault, Joerg Huelsken, Elisabeth Brambilla, Stanislas du Manoir

**Affiliations:** 1 IGBMC (Institut de Génétique et de Biologie Moléculaire et Cellulaire); Département Biologie du Cancer, INSERM (Institut National de la Santé et de la Recherche Médicale), U964, Illkirch, France; 2 IGBMC (Institut de Génétique et de Biologie Moléculaire et Cellulaire); Département Biochip, INSERM (Institut National de la Santé et de la Recherche Médicale), U964, Illkirch, France; 3 CNRS (Centre National de la Recherche Scientifique), UMR 7104, Illkirch, France; 4 Université de Strasbourg, Strasbourg, France; 5 Collège de France, Chaire de Génétique, Illkirch, France; 6 INSERM, U728, Hôpital Saint-Louis, Paris, France; 7 EPFL SV ISREC CDTSO (Institut Suisse de Recherches Expérimentales sur le Cancer–Ecole Polytechnique Fédérale de Lausanne), Lausanne, Switzerland; 8 Institut Albert Bonniot, Département Oncogénèse et Biotechnologie, INSERM, U578, La Tronche, France; University of Missouri Kansas City, United States of America

## Abstract

Squamous cell carcinoma (SCC) of the lung is a frequent and aggressive cancer type. Gene amplifications, a known activating mechanism of oncogenes, target the 3q26-qter region as one of the most frequently gained/amplified genomic sites in SCC of various types. Here, we used array comparative genomic hybridization to delineate the consensus region of 3q26.3 amplifications in lung SCC. Recurrent amplifications occur in 20% of lung SCC (136 tumors in total) and map to a core region of 2 Mb (Megabases) that encompasses *SOX2*, a transcription factor gene. Intense SOX2 immunostaining is frequent in nuclei of lung SCC, indicating potential active transcriptional regulation by SOX2. Analyses of the transcriptome of lung SCC, SOX2-overexpressing lung epithelial cells and embryonic stem cells (ESCs) reveal that SOX2 contributes to activate ESC-like phenotypes and provide clues pertaining to the deregulated genes involved in the malignant phenotype. In cell culture experiments, overexpression of SOX2 stimulates cellular migration and anchorage-independent growth while SOX2 knockdown impairs cell growth. Finally, SOX2 over-expression in non-tumorigenic human lung bronchial epithelial cells is tumorigenic in immunocompromised mice. These results indicate that the *SOX2* transcription factor, a major regulator of stem cell function, is also an oncogene and a driver gene for the recurrent 3q26.33 amplifications in lung SCC.

## Introduction

Recurrent chromosomal aberrations represent driving forces of tumor progression and historically have led to the identification of tumor suppressor genes and oncogenes critical for several cancer types [1]. Gene copy number increases, such as gene amplifications, are well-known oncogene activating mechanisms, affecting their expression by gene dosage [2], [3]. Amplification of canonical oncogenes, such as *EGFR*, *ERBB2, C-MYC*, *N-MYC*, *CCND1, MDM2* and *RAS*, have been found in 10 to 30% of cases of the different cancers types in which they play a central role [2]. In squamous cell carcinomas (SCCs), chromosome 3 aberrations are among the most prevalent. Recurrent deletions on the 3p arm and gains on the 3q arm are present in the majority of cases, whereas gene amplifications cluster in the 3q26–q29 region [4], [5]. Several amplicon units at 3q26-qter have been postulated to be implicated in the disease [6], [7]. At 3q26.3, the *DCUN1D1* gene has been recently proposed as an oncogene in lung SCC [8]. Nevertheless, the precise location and frequency of 3q26.3 amplifications in lung SCC remain unclear. In addition, more than one oncogene can be co-activated within an amplicon and synergistically participate in different tumor traits, as exemplified for another locus in lung adenocarcinoma [9]. Therefore, precise mapping of the amplification region and firm demonstration of oncogenic properties of individual genes from the amplicon will permit assessment of their relative contribution to the tumor phenotype.

To delineate regions of chromosome 3 alterations with precision, we screened genomic unbalances in 26 lung SCCs using high-resolution dedicated arrays. We selected operable locally advanced (stage III) lung SCC because advanced tumors have a tendency to contain more genomic aberrations, including gene amplifications, selected during tumor progression [10]. We uncovered a core amplified region of 2 Mb at 3q26.33 containing nine genes, which include *DCUN1D1* and the transcription factor *SOX2*.

SOX2 (abbreviation of sex-determining region Y-box 2) is a 317 aminoacid transcription factor containing an HMG domain and a critical transcription regulator of normal stem cell function in embryonic and neural stem cells [11]–[13]. SOX2 is a major stemness factor. Indeed, it is a critical transcription regulator of the normal stem cell phenotype of ESCs, with a restricted number of partners, including Oct-4 and Nanog. It controls self-renewal and differentiation processes through coordinated transcriptional programs [11], [12]. Likewise, SOX2 is a major regulator of stem cell function in NSCs (Neural Stem Cells) [13]. SOX2 is one of the “magical four” crucial transcription factors capable of cooperating to reprogram differentiated cells into an induced pluripotent stem cell-like phenotype [14], [15].

In murine lung development, SOX2 controls branching morphogenesis and epithelial cell differentiation, and its over-expression leads to an increase in committed precursor-like cells, notably basal cells [16]. In all of these individual situations, SOX2 modulates coordinated transcriptional programs that are dependent on available cofactors in the cellular context.

Here, we show that *SOX2* has a major impact on global lung SCC transcriptome deregulation and contributes to activate ESC-like transcriptome phenotypes, thereby establishing SOX2 as a key up-regulated transcription factor in lung SCC which modulates both direct and indirect key target genes involved in tumor progression. Over-expression in human lung epithelial cell grafts in immunocompromised mice led to the formation of poorly differentiated squamous tumors with basaloid traits. Together, our work identifies SOX2 as an oncogene and likely driver gene of one of the most frequent amplification sites in lung SCC.

## Results

### Array Comparative Genomic Hybridization Screening for Chromosome 3 Aberrations in Lung SCC

**Figure 1:**
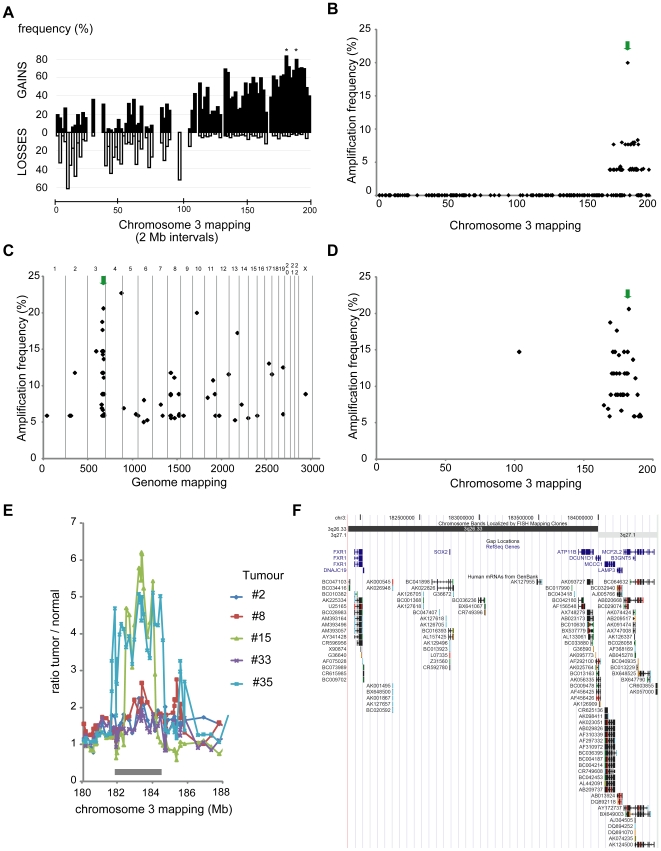
Characterization of chromosome 3 aberrations in lung SCC using array-CGH. **A.** Frequency of chromosome 3 losses and gains in 26 advanced lung SCCs. For each 2 Mb interval along chromosome 3, frequencies were calculated by dividing the total number of occurrences of losses or gains observed in the series by the total number of possible occurrences, expressed as percentages and represented along the chromosomal order. Gaps are due to intervals with no coverage on the array. Frequent 3p losses seem to affect discontinuous zones. The most frequently lost region is the 8–10 Mb interval (around 60% of samples). Gains were more frequently detected on the 3q arm: 3q26-qter was gained in 50–60% of the samples with two discrete intervals (180–182 and 188–190 Mb, denoted by asterisks) gained in around 80% of tumors. **B.** High level chromosome 3 amplification frequency in 26 advanced lung SCCs. For a given clone, a ratio greater than 2 (1 in log2 scale) in a single experiment was considered to represent a high-level amplification. Frequencies were calculated for the series of 26 cases and plotted according to clone localization. High level amplifications cluster in the 3q26-qter region. A peak maxima is observed for BAC clone RP11-259I19; high level amplifications of this locus are found in 20% of cases (5/26 tumors). **C.** Whole genome high level amplification frequencies in 34 advanced lung SCCs (independent cohort). Chromosome numbers are indicated above the graph, and different chromosomes are separated by vertical gray lines. For a given clone, a ratio greater than 1.5 in log2 scale in a single experiment was considered to represent a high-level amplification. Frequencies were calculated for the series of 34 cases and plotted according to clone localization on the genome. Two genomic regions, on chromosomes 3 and 4 were amplified in >20% of the cases. **D.** High level chromosome 3 high level amplification frequency in 34 advanced lung SCCs (independent cohort). The graph corresponds to panel C restricted to chromosome 3. **E.** Individual array-CGH profiles obtained for the five tumors with high level 3q26.3 gene amplifications. High ratio deviations were observed for two tumors with straight amplicon boundaries (#15 and #35). The consensus region of 3q26.3 amplifications is a 2.7 Mb segment spanning the 181.9–184.6 Mb interval (denoted by a grey rectangle). **F.** UCSC genome browser map of the 3q26.33 consensus region of amplifications in lung SCCs. This fully sequenced and assembled genomic region (181.9–184.6 Mb, NCBI build 34) contains nine genes (Refseq), including *DCUN1D1* and *SOX2*, and various Genbank mRNAs. This image was downloaded from the UCSC genome browser (http://genome.ucsc.edu/, [64], [65]). The green arrows in panels B and C/D point to BAC clones RPCI11-259I19 and RPCI11-701O19, respectively, corresponding to the maxima of chromosome 3 amplification in these lung SCC cohorts. RPCI11-259I19 and RPCI11-701O19 are mapped, respectively, between the *SOX2* and *DCUN1D1*, and *FXR1* and *SOX2,* genes. Two different clones were found due to the composition of the two arrays (RPCI11-259I19 in our Chr3 array and RPCI11-701O19 in the GSE12280 array). Mapping of the amplification by two independent clones suggest that it is not a clone-derived artifact.

To delineate chromosome 3 consensus regions of deletions and gains/amplifications, we analyzed 26 advanced stage lung SCCs using a chromosome 3-dedicated array composed of 214 genomic clones. All data are available in GEO (GSE15080). Losses on the 3p arm, gains of large 3q regions and high-level amplifications at 3q26-qter were found (Figure 1 panel A–B). Deletions mostly occurred on the 3p arm and seemed to affect several sites. Among genomic positions evaluated on the short arm, the interval from 8 to 10 Mb, (containing *OGG1* and *RAD18)* was the most frequently lost (60% of tumors). Large gains often targeted the 3q arm, with a global gain of the 3q26-qter (176–196 Mb) region in 60% of tumors. Two interval regions, from 180 to 182 Mb (including the *PIK3CA* locus) and from 188 to 190 Mb (including the *RFC4/SST* locus) were gained in about 80% of tumors. Frequent high-level amplifications clustered in the 3q26-qter region (Figure 1 panel B), with a maximum for clone RPCI11-259I19 amplified in nearly 20% (5/26). This clone is located at 3q26.33, between *SOX2* and *DCUN1D1* (Figure 1 panel F). Whole genome surveys of the two tumors with the most accentuated amplifications revealed that the highest copy number levels across all chromosomes were located at 3q26.33 (Figure S1, panel A).

To estimate the general relevance of these findings, we explored pangenomic array-CGH data in additional and independent cohorts of SCCs from the lung or uterine cervix. We found consistent results with the most common amplification at the same locus in a second independent cohort of 76 lung SCCs (N. Martinet and S. du Manoir, unpublished data). In addition, in a third independent cohort (34 lung SCCs, GSE12280, [17]), two genomic regions are amplified recurrently (>20% of the tumors), including the 3q26.33 locus represented by the clone RP11-701O19 (Figure 1 panel C–D). This clone maps between the *FXR1* and *SOX2* genes (Figure 1panel F ). Furthermore, in uterine cervix SCCs (GSE6473, [18]; GSE11573, [19]), this locus is the most frequently amplified on chromosome 3 (4 to 10% of cases, Figure S1 panel B). In conclusion, the 3q26.33 locus undergoes copy number increase in a high percentage of lung SCCs (60 to 80%), and high-level amplifications recurrently occur in 20% of the tumors in three independent cohorts (>130 lung SCCs in total). Finally, this site is one of the most commonly affected in uterine cervix SCC, showing that 3q26.33 is a recurrently altered locus in SCCs and likely involved in their pathogenesis.

### High-Resolution Mapping of Recurrent 3q26.33 Amplicons in Lung SCC

To refine mapping of the 3q26.33 amplifications, we built a 3q26.3–3q27 tiling array (from 177 Mb to 186 Mb). Re-analysis of the five tumors with amplifications confirmed the presence of 3q26.33 amplicons in each case and allowed us to delineate their size exemplified for two cases (Figure S1 panel C–D). The core amplicon in these tumors was a segment of slightly less than 2 Mb in size, mainly covering the 3q26.33 band and a small segment of 3q27.1 (Figure 1 panel E–F). We listed the gene content in a slightly larger region of 2.7 Mb (from 181.9 to 184.6 Mb) to take into account the high-level gains of neighboring regions. Nine known genes were included in this genomic interval and stand as oncogene candidates to be activated by recurrent 3q26.33 amplifications (Figure 1 panel F).

### Candidate Genes Expression Levels and 3q26.33 Copy Number Changes in Lung SCC

**Figure 2:**
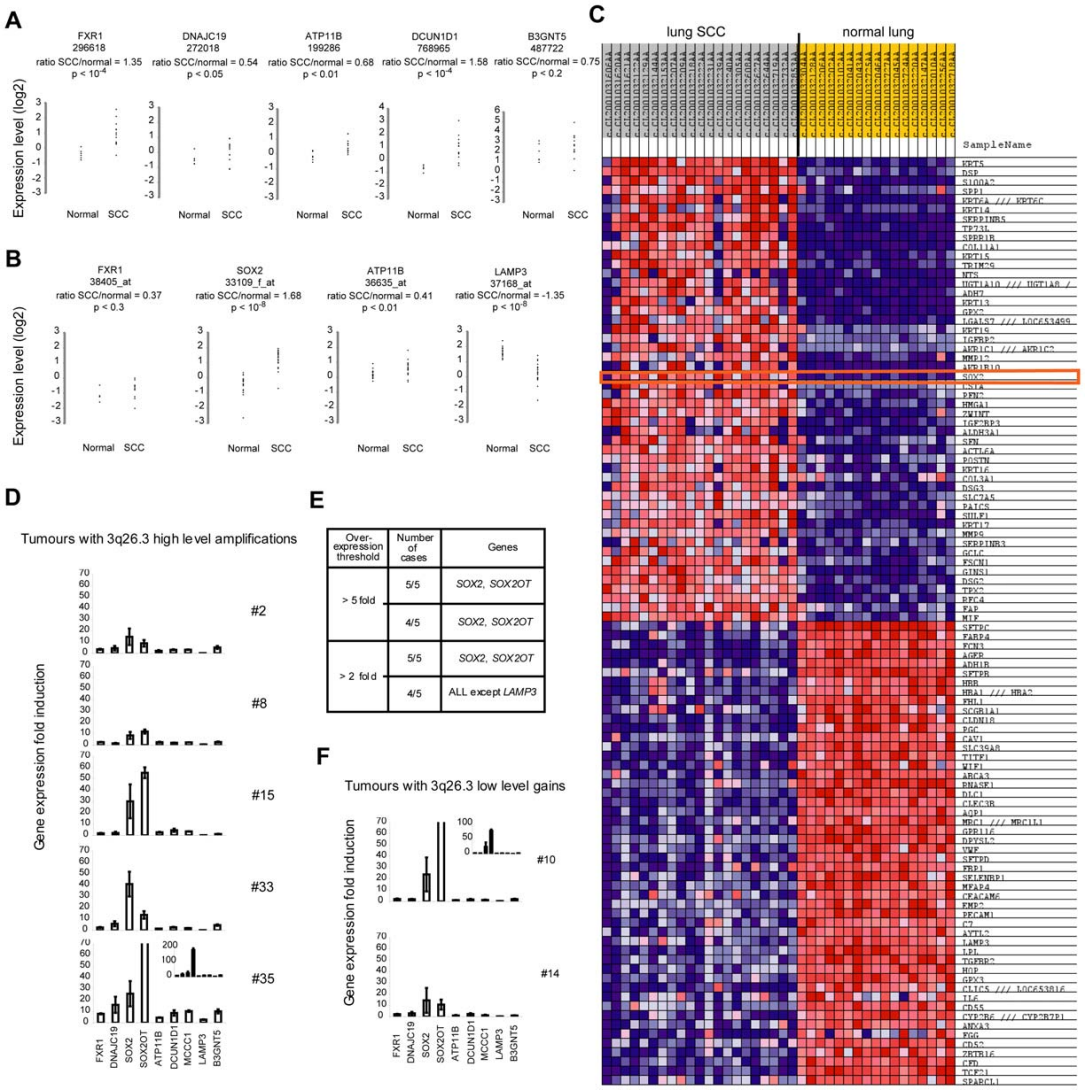
Transcriptional consequences of 3q26.33 copy number increases in lung SCC. Panels A and B: expression levels of 3q26.33 genes in lung SCCs versus normal comparisons using microarrays and Oncomine. Each graph represents normalized log2 expression values of a given gene in normal lung and lung SCC samples. The clone (panel A) or Affymetrix probe set (panel B) number is indicated under the gene name. The log2 value ratio (average expression value in SCC)/(average expression value in normal lung) and the corresponding p-value are indicated. **A.** Lung SCC dataset-2. Among the five genes that were analyzable and represented (*FXR1*, *DNAJC19*, *ATP11B*, *DCUN1D1*, and *B3GNT5*), *FXR1*, *ATP11B*, and *DCUN1D1* are significantly over-expressed in lung SCCs (all with p<10^−2^). **B.** Lung SCC dataset-1. Four genes localized in the 3q26.3 core amplicon are represented on the microarray (*FXR1*, *SOX2*, *ATP11B*, and *LAMP3*). *LAMP3* is strongly down-regulated in the tumors (p<10^−8^). *ATP11B* (p<10^−2^) and *SOX2* (p<10^−8^) are significantly over-expressed in lung SCCs. **C.** Top 100 genes deregulated in lung SCC dataset-1 using GenePattern. These genes include *SOX2* (red rectangle) among the top 50 genes over-expressed in lung SCCs compared to normal lung. In this dataset, four genes (*FXR1*, *SOX2*, *ATP11B*, and *LAMP3*) out of the nine localized in the core amplicon were represented on the array. For each gene in each sample, expression is represented by a square with a color that codes for the level as a gradient (dark blue, low expression, to dark red, strong expression). **D.** Expression levels of nine genes from the consensus region in the tumors with high level 3q26.3 amplifications. The identical y-scale for all graphs represents the expression ratio for a given gene when compared to its relative average expression level in two lung SCCs without copy number change of the 3q26.3 locus. Genes are ranked from left to right according to 3q26.33 genomic localization, and names are represented below the bottom graph. **E.** Summary of over-expressed genes in the five lung SCCs with high-level 3q26.3 amplifications. Two genes, *SOX2* and *SOX2OT*, are consistently over-expressed in tumors with 3q26.3 copy number increases when compared to tumors with a normal copy number of the locus. Other genes are recurrently over-expressed but at lower levels when compared to *SOX2* and *SOX2OT*. **F.** Two tumors with low-level copy number gains of the 3q26.3 locus were analyzed as in panel D. *SOX2* and *SOX2OT* are the most over-expressed genes in these tumors. Panels D and F: Sample numbers are indicated on the right of the graphs. Insets in tumor #35 and #10 graphs correspond to y-axis extended scale to fold-change values of 200 and 100, respectively. Fold-changes represent the mean ± sem of three independent experiments.

To determine the extent to which the nine candidate genes at 3q26.33 were differentially expressed in lung SCC, we analyzed published, genome-wide transcriptome datasets [20], [21] through Oncomine [22] (Figure 2 panel A–B). *LAMP3* expression is greatly down-regulated, whereas *FXR1*, *ATP11B, DCUN1D1* and *SOX2* are significantly up-regulated in primary tumors compared to normal lung. Importantly, *SOX2* is over-expressed in about 90% of the tumors and is among the top 50 genes (rank 21) most strongly over-expressed in lung SCC, together with several classical squamous markers, such as keratins and p63 (Figure 2 panel C and Table S1). To discriminate between driver and by-stander genes within the 3q26.3 core amplicon, we next investigated the expression levels of the nine candidate genes in the five tumors with 3q26.3 genomic amplifications, in comparison to tumors with low level gains or with normal copy number, using RT-qPCR. In the five tumors with amplifications, all of the genes, with the exception of *LAMP3,* were recurrently over-expressed (four out of five tumors with an expression ratio >2) except *LAMP3* (Figure 2 panel D and E). However, recurrent strong over-expression (four out of five tumors with a ratio >5) is found only for *SOX2* and *SOX2OT*. In the two tumors with low-level gains (Figure 2 panel F), the two highly up-regulated genes were also *SOX2* and *SOX2OT*. In conclusion, eight out of the nine tested genes were recurrently over-expressed, but *SOX2* and *SOX2OT* were the most consistently highly over-expressed and thus are the best candidates to be driver genes of 3q26.3 amplifications.

### Immunohistochemical Analysis of SOX2 in 51 Human Lung SCCs

**Figure 3:**
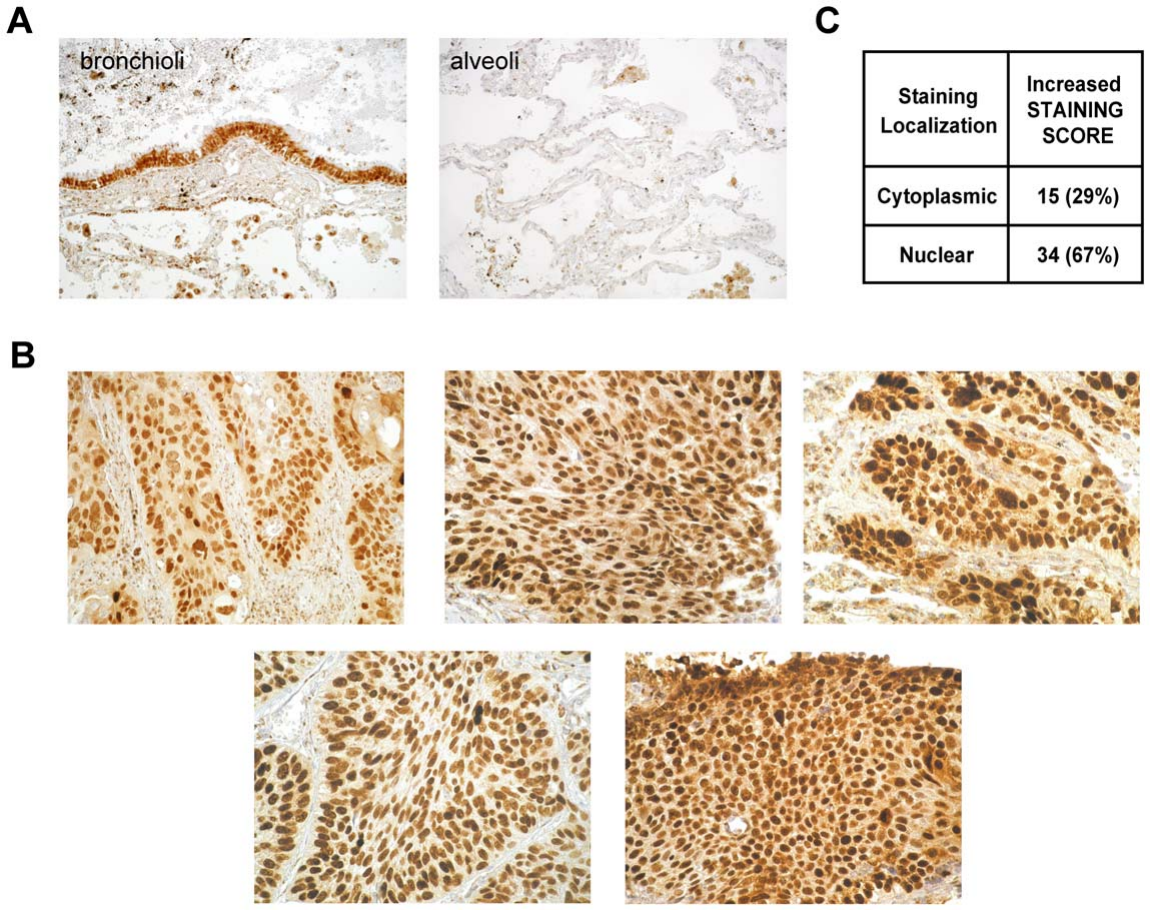
SOX2 immunohistochemical analysis in 51 advanced lung SCCs. **A.** Sox2 staining pattern in a normal human lung sample (left panel: bronchioli; and right panel: alveoli). SOX2 is detected at a low level in the cytoplasm of all bronchial and bronchiolar epithelial cells, and staining intensity is strong in the nucleus of 30% of these cells. Alveolar epithelial cells are negative. **B.** Sox2 staining in the five tumors with high-level 3q26.33 amplifications. SOX2 is strongly expressed in the nucleus of most tumor cells (>80%). **C.** Summary of staining scores for the 51 lung SCCs. Staining was scored according to intracellular localization (cytoplasmic or nuclear) and intensity. For each category, the number of cases is indicated, and the corresponding percentage (over the series of 51 samples) is indicated between brackets. SOX2 activation, as defined by stronger nuclear staining and detection in a higher proportion of cells when compared to normal lung epithelial cells, is observed in a wide majority of lung SCCs (67%). All pictures of tumors were acquired at 250x magnification.

We investigated SOX2 protein expression by immunohistochemistry in 51 lung SCCs (these cases included the 26 advanced stage lung SCCs studied by array-CGH). Staining was scored according to intracellular localization, intensity and the percentage of positive cells. Individual scoring results for the 51 samples are presented in Table S2. In the normal human lung, SOX2 staining was mainly observed in bronchial epithelial cells (but not alveolar cells, Figure 3 panel A), where nuclear SOX2 expression was restricted to a sub-population (30%) of bronchial epithelial cells. In tumors, a few cases incidentally harbor strong and exclusively cytoplasmic staining (five cases, 10%), and the five cases with 3q26.33 amplifications display strong nuclear staining in nearly all tumor cells (>80%) with the highest signal scores (Figure 3 panel B). Overall, 34/51 tumors (67%) have an increased nuclear Sox2 staining score (Figure 3 panel C). In conclusion, the Sox2 transcription factor is strongly expressed and localized in the nucleus in the wide majority of lung SCCs, showing that Sox2 is recurrently activated and suggesting that it may consequently regulate a coordinated and specific transcriptional program in lung SCC.

We thus wondered whether Sox2-dependent expression signatures could be detected in these tumors. We analyzed two independent human lung SCC genome-wide transcriptome datasets (dataset-1 [20]; dataset-2 [21]) and defined lung SCC signatures (genes that were differentially expressed in lung SCC vs. normal comparison, see Genelist S1). Our objectives were: first, to comprehensively characterize lung SCC molecular phenotypes at the transcriptome level; and second, to assess the contribution of SOX2. Previous studies have clearly established that molecular signature comparisons are more robust when carried out at an integrative level to reveal their relative enrichments [23], [24], using a knowledge-based and/or concomitantly expressed group of genes, such as gene modules [25]. These powerful approaches condense independent and multiple experimental observations into definite modules of genes that are coordinately activated or repressed in a large compendium of experiments.

### Stem Cell-Like Transcriptome Phenotypes in Human Lung Squamous Cell Carcinoma

**Figure 4:**
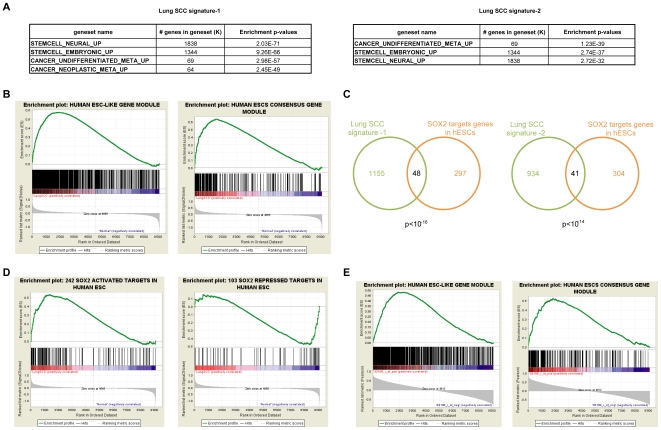
A global SOX2 imprint on the lung SCC transcriptome towards an embryonic stem cell-like molecular phenotype. **A.** Selected molecular signatures enriched in the lung SCC signatures −1 and −2. For each signature, enrichments were queried using the molecular signatures database. Highly significant enrichment of both embryonic and neural stem cell-specific molecular signatures was observed in the lung SCC transcriptome (all with p<10^−31^, hypergeometric distributions). **B.** Enrichments of ESC-like gene modules with known relevance for epithelial cancers in lung SCCs. The GSEA tool was used to query enrichments of the human ESC-like (left panel) and the human ESC consensus (right panels) gene modules in the lung SCC dataset-1. Significant enrichments are observed in lung SCCs (both FDR <0.02). **C.** Venn diagrams of the overlaps between the 345 SOX2 target genes in hESCs and the lung SCC signatures −1 (left graph) or −2 (right panel). Gene numbers are indicated within the corresponding sections, and the p-value is below each graph (hypergeometric distribution). **D.** Enrichments of known SOX2 target genes in lung SCCs (dataset-1). The GSEA tool was used to query enrichments of SOX2 target genes that are activated (left panel) or repressed by SOX2 (right panel) in human ESCs. Significant enrichment of SOX2-activated target genes is observed among genes over-expressed in lung SCCs (FDR <0.03) and of SOX2- repressed genes among genes under-expressed in lung SCCs (FDR  = 0.13). **E.** Enrichments of ESC-like gene modules with known relevance for epithelial cancers among genes correlated to SOX2 expression in lung SCCs. The GSEA tool was used to query enrichments of the human ESC-like and the human ESC consensus gene modules in the lung SCC dataset-1 using SOX2 expression to define phenotypes. Significant enrichments are observed (both FDR <0.05).

Accordingly, we searched for enrichments of the lung SCC signatures in the Molecular Signatures Database (see Text S1). We found enrichments of a meta-signature of poorly differentiated human cancer [26] (Figure 4 panel A). More surprisingly, we also uncovered highly significant enrichments of ESC and NSC signatures in the lung SCC signatures. To follow this clue, we retrieved compiled ESC-like signatures recently issued in two large scale meta-analyses [27], [28]. From a large and curated compendium of stem cell profiling experiments, an ESC-like gene module was isolated and shown to be activated in several human epithelial tumor types. Its activation correlates with poor prognosis in breast and lung adenocarcinoma [28]. Similarly, a meta-analysis [29] has permitted the compilation of a human ESC consensus gene module that is activated in poorly differentiated, high grade breast, glioblastoma and bladder cancers [27]. We addressed the question of the similarity between the lung SCC transcriptome and these ESC-like molecular signatures. Both the hESC consensus and the hESC-like modules were highly significantly enriched in primary SCC (all FDR <0.02, 23-34% of the modules; Figure 4 panel B and Figure S2 panel A, see Genelist S1). Our findings show that stem cell-like molecular phenotypes are activated in lung SCC, and the frequent nuclear staining observed in tumors is consistent with a SOX2 transcriptional regulation function, leading us to further dissect the impact of SOX2.

### SOX2 Contribution to Stem-Cell Like Transcriptome Phenotypes in Lung SCC

Promoter analyses revealed over-representation of SOX proteins binding sites in the lung SCC signatures (not shown), consistent with an imprint of SOX2 activity on these transcriptomes. SOX2 is a major regulator of stem cell function in both ESCs and NSCs, controlling SC fate and self renewal. Accordingly, SOX2 targets have been extensively identified in this cellular context. We intersected the lists of genes that are differentially expressed [29] and have SOX2-bound promoters in ESCs [11] to generate a curated list of 345 known direct SOX2 target genes in human ESCs (242 and 103 genes that SOX2 activates or represses, respectively, see Genelist S1). We compared this list of known SOX2 targets to the lung SCC signatures and found significant overlaps (Figure 4 panel C). In fact, approximately 4% of the genes comprising the lung SCC signatures are known SOX2 targets in hESCs. This enrichment was further confirmed by GSEA-based analyses (Gene Set Enrichment Analysis, Figure 4 panel D and Figure S2 panel B). For comparison, we performed identical analysis for the *c-Myc* oncogene with a curated list of 415 known *c-Myc* target genes and uncovered similar proportions (5.2% and 3.2% of deregulated genes in lung signatures -1 and -2, respectively). To conclude, a significant proportion of direct SOX2 target genes are deregulated in lung SCC to an extent comparable to a well-established oncogenic transcription factor. This finding is strongly in favor of a notable SOX2 impact on the lung SSC transcriptome.

To address the question of whether SOX2 contributes to the stem-cell like molecular traits of lung SCC, we performed transcriptome analyses of an *in vitro* cellular model, and we also explored genes with expression profiles that correlate with SOX2 in primary tumors (GSEA-based correlative analyses). We created a simple *in vitro* cellular model of human bronchial epithelial cells by stably over-expressing SOX2 in the BEAS-2B immortalized and non-tumorigenic cell line [30]. We found 922 differentially expressed genes between Sox2-transduced and control cells that comprise the SOX2-squamous signature (see Genelist S1). As expected, the promoters of these genes were enriched with SOX protein binding sites (p<10^−6^). Additionally, the SOX2-squamous signature in BEAS-2B cells significantly overlaps with the known direct SOX2 targets defined in hESCs. Moreover, this signature is enriched in several GO categories (Figure S3 panel A–C). More interestingly, SOX2 over-expression in this *in vitro* model led to activation of a significant proportion of genes from the human ESC-like gene module (Figure S3 panel D; FDR  = 0.12, 292 genes, 23% of the module). Finally, to further assess the contribution of SOX2 to the activation of ESC-like modules in primary tumors, we performed correlative analyses using GSEA. Genes with expression profiles that correlated with SOX2 expression in the lung SCC datsaset-1 (no valid SOX2 probe is present in dataset-2) were found to be significantly enriched for the hESC consensus (FDR <10^−2^) and hESC-like (FDR <0.07) modules (Figure 4 panel E). These results demonstrate that human lung SCCs have marked ESC-like molecular phenotypes: 23 to 34% of the genes from ESC-like modules, which were shown to be activated in aggressive epithelial tumors, are consistently expressed in human lung SCC. In primary lung SCC, as well as in *in vitro* over-expressing cells, SOX2 is capable of modulating a significant portion of the hESC-like module. Altogether, these findings strongly argue for a direct contribution by SOX2 to these ESC-like molecular traits in lung SCC *in vivo*. The next question that we considered was the possible functional consequences over-expression of SOX2 on lung squamous cell carcinomas.

### Consequences of SOX2 Over-Expression on Lung SCC Malignant Phenotype

**Figure 5:**
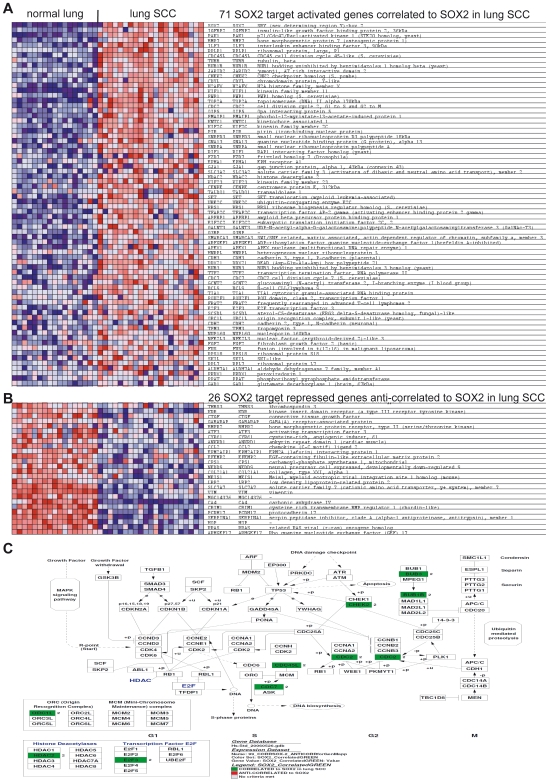
List of known direct SOX2 targets in hESCs that significantly correlate and anti-correlate to with SOX2 expression in lung SCCs. **A.** The 71 genes that are known SOX2-activated target genes in human ESCs and significantly correlate with SOX2 expression in lung SCCs. **B.** The 26 genes that are known SOX2-repressed target genes in human ESCs and significantly anti-correlate with SOX2 expression in lung SCCs. Panels A and B: For each gene in each sample, expression is represented by a square with a color that codes for the level as a gradient (dark blue, low expression, to dark red, strong expression). **C.** Genmapp view of the enriched cell cycle regulators that are targets of SOX2 in hESCs and correlate with SOX2 expression in lung SCCs. Green boxes represent the genes that are significantly enriched among the known SOX2-activated target genes in human ESCs and correlate to SOX2 expression in lung SCCs.

To identify likely direct targets of SOX2 in lung SCC, we analyzed the correlation/anti-correlation between SOX2 expression levels and the known SOX2 direct target genes in the tumors. Among known SOX2-activated targets, 71 genes significantly correlated with SOX2 expression and were over-expressed in lung SCC (Figure 4 panel E, FDR <10^−2^). Known SOX2-repressed direct targets were relatively enriched as well, but as expected, they were among genes anti-correlated to SOX2 expression in the tumors (Figure 4 panel E, FDR  = 0.13). Together, they form a list of 97 known SOX2 target genes directly deregulated downstream of SOX2 in lung SCC (Figure 5 panel A and B, Table S3. This list is significantly enriched in cell cycle genes, a meta-signature of poor differentiation in cancer and embryonic and neural SC-specific gene modules (not shown). The over-represented cell cycle genes include several crucial regulators of cell cycle progression, such as *CDC2*, *CDK1*, and *E2F3* (Figure 5 panel C), suggesting that SOX2 over-expression contributes to perturbation of the cell cycle in primary lung SCC. To take into account indirect gene deregulations downstream of SOX2, we computed all overlaps between the SOX2-squamous *in vitro* model and the two lung SCC signatures (present in SOX2-squamous and in any of the lung SCC signatures). This process resulted in a consolidated list of 142 genes (Table S4). The ECM-receptor interaction pathway was significantly enriched among up-regulated genes, comprising collagens 1A1 and 3A1 and Integrins β1 and β8. Known TSGs (Tumor Suppressor Gene) and apoptosis inducers (Trail, Caspases 1 and 4) were found to be down-regulated and several transcription factors, secreted signaling molecules and a RAS-like oncogene (Rit1) were up-regulated. All of these genes stand as sound candidates as they are downstream of SOX2 and have the potential to impact lung SCC carcinogenesis. Among them (genes from Table S4), we selected 16 outstanding candidate genes selected for their strong relevance to cancer biology, as indicated by the scientific literature (Table 1).

**Table 1:** Selection of 16 cancer-related genes as potential mediators of the malignant phenotype of lung SCCs downstream of SOX2.

	Gene Symbol	Gene title	Comment
	CBX5	chromobox homolog 5 (HP1 alpha homolog, Drosophila)	Cooperates with Myb and Hmgb3 to form ESC-like myeloid Leukemic Stem Cells.
	GATA3	GATA binding protein 3	Transcription factor, poor prognosis marker in endometrial and breast cancers.
	MYCL1	v-myc myelocytomatosis viral oncogene homolog 1, lung carcinoma derived (avian)	L-Myc oncogene.
	HOXB7	homeobox B7	In breast cancer, promotes EMT, the angiogenic switch, tumor progression and lung metastasis.
	HOXC4 /// HOXC6	homeobox C4 /// homeobox C6	Over-expressed in primary prostate tumors and lymph node metastases
	RIT1	Ras-like without CAAX 1	Ras-like transforming oncogene.
	IGFBP5	insulin-like growth factor binding protein 5	Pro-fibrotic factor in lung, induces EMT. Prognostic value in breast cancer.
	NRG1	neuregulin 1	EGFR/ERBBs ligand, induces lung branching morphogenesis through PI3K pathway and lung epithelial cells proliferation.
	FGFBP1	fibroblast growth factor binding protein 1	Mobilizes and activates stored FGFs to induce the angiogenic switch in tumors.
	FHL1	four and a half LIM domains 1	Tumor suppressor gene
	PTGIS	prostaglandin I2 (prostacyclin) synthase	Over-expression prevents lung carcinogenesis in mice model. Anti-inflammatory and anti-proliferative roles.
	CASP1	caspase 1, apoptosis-related cysteine peptidase (interleukin 1, beta, convertase)	Apoptosis inducer
	CASP4	caspase 4, apoptosis-related cysteine peptidase	Apoptosis inducer
	CLU	clusterin	Absence of Clusterin in NSCLC is associated to a shorter survival
	TGFBR3	transforming growth factor, beta receptor III	Suppresses NSCLC cells invasion and tumorigenicity.
	TNFSF10	tumor necrosis factor (ligand) superfamily, member 10	TRAIL, Apoptosis inducer

Deregulated genes in lung SCC signatures and the SOX2-squamous signature (from Table S4) were selected based on their known function related to cancer or correlation to patient prognosis in the scientific literature.

**Figure 6:**
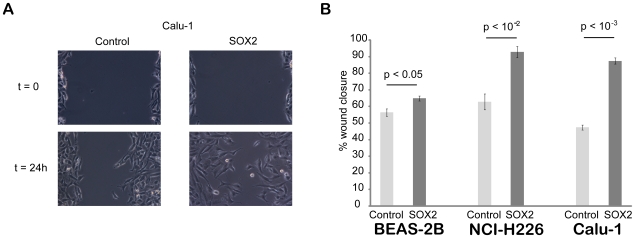
Consequences of Sox2 over-expression in the wound healing *in vitro* assay. **A.** Pictures were acquired at the beginning of the experiment (t = 0, immediately after wounding) and from the same field at the end of the experiment (t = 24 h for this example from the Calu-1 cell line). **B.** Quantification of wound closure for the three cell lines (BEAS-2B, NCI-H226 and Calu-1). Wound sizes were measured at the beginning and end of the experiment to calculate the percentage of wound closure for control and SOX2 over-expressing cells for each of the three cell lines. SOX2 over-expression significantly stimulates cell migration compared to control cells (student's t-test).

To perform functional analyses of the consequences of SOX2 over-expression, we established three human lung epithelial cell lines stably over-expressing SOX2 (Figure S4). Noting several cell cycle genes in the above-mentioned gene lists, we initially checked the proliferation rate of these cell lines *in vitro,* but uncovered no differences (data not shown). We therefore tested their migratory and invasive capacities. While no significant difference was observed for cell invasion (data not shown), SOX2 over-expression significantly increased the migratory activity of the three cell lines as assessed using a wound healing *in vitro* assay (Figure 6). We next investigated the effects of SOX2 over-expression on anchorage-independent growth of BEAS-2B cells using the soft-agar colony formation assay. While BEAS-2B control cells gave rise to some small colonies, as previously reported [31], SOX2 over-expression induced a significant increase in the number of colonies that were much larger and became macroscopically visible (Figure S5), which is a hallmark of malignant cells [32]. These results indicate that SOX2 over-expression significantly stimulates BEAS-2B cell anchorage-independent growth and suggests that SOX2 could represent a transforming oncogene when over-expressed in lung epithelial cells.

**Figure 7:**
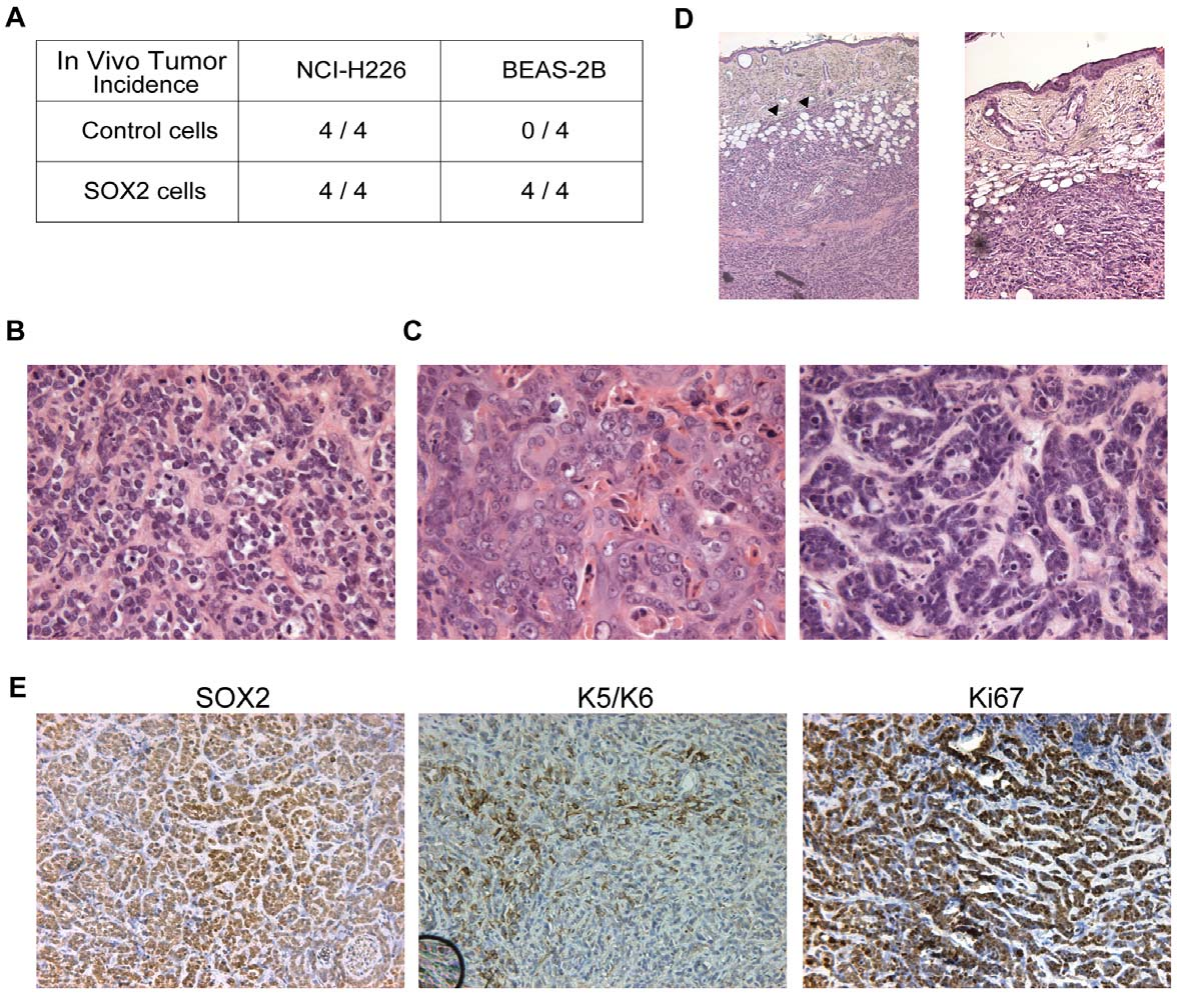
Consequences of Sox2 over-expression *in vivo*. **A.** Tumor incidence upon subcutaneous implantation of human lung squamous control- and SOX2-transduced cell lines in nude mice. For each cell line, the number of tumors that developed is represented with respect to the number of injections for each cell type (control or SOX2-transduced, n = 4 injected animals each). Tumor incidence is unchanged for the NCI-H226 highly tumorigenic cell line, whereas BEAS-2B cells become tumorigenic upon Sox2 over-expression. **B.** Hematoxylin and Eosin (H&E) staining of a representative area of a BEAS-2B-Sox2 subcutaneous tumor (magnification = 100×). A majority of the tumor area (around 80%) has typical traits of poorly differentiated basaloid variants of squamous cell carcinoma. **C.** H&E staining of representative areas of the same BEAS-2B-Sox2 subcutaneous tumor as in panel B (magnification = 100×). Around 20% of the tumor area has typical traits of poorly to moderately differentiated squamous cell carcinoma, with individual cell keratinization. **D.** H&E staining of one BEAS-2B-Sox2 subcutaneous tumor (magnification = 50×). In this case, local tumor cell invasion into the dermis was observed (arrowheads). **E.** Immunohistochemistry for SOX2, Keratins 5/6 and Ki67 (left, middle and right panels, respectively; magnifications = 200×). Tumors homogeneously express SOX2 and Ki67 and heterogeneously express Keratins 5/6, which are squamous cell differentiation markers.

Therefore, we implanted transduced cells *in vivo* to assess the effect of Sox2 over-expression on tumor formation and growth in immunocompromised mice injected subcutaneously. The highly tumorigenic NCI-H226 cells lead to systematic tumor growth with no change in growth rate according to SOX2 status (Figure 7 panel A). By contrast, BEAS-2B-SOX2 cells gave rise to tumors in all four injected animals, in contrast to control cells (Figure 7 panel A–D). The BEAS-2B-SOX2-derived tumors grew relatively slowly: volumes reached 0.25–1 cm^3^ within 3 to 6 months. This finding strongly suggests that additional hits downstream of SOX2 over-expression might be required to allow for full transformation of BEAS-2B cells and tumor expansion *in vivo*. At the histological level (Figure 7 panel B), the tumors were mainly composed of poorly differentiated SCC of the basaloid subtype [33]. Remarkably, within the same tumor, restricted areas (about 20% of the tumor section surface) displayed well- or moderately-differentiated features with individual cell keratinization (Figure 7 panel C), which is commonly seen in human basaloid variants of SCC [34]. Interestingly, local invasion of tumor cells into the dermis was also noted in one case (Figure 7 panel D). At the molecular level, low levels of apoptosis were noted (as assessed by cleaved caspase-3 staining; data not shown). The tumor cells homogeneously expressed nuclear SOX2, and most of them were cycling, as assessed by Ki67 staining. Further, keratins 5/6, which are squamous differentiation markers, were heterogeneously expressed (Figure 7 panel E). In conclusion, Sox2 over-expression is capable of converting non-tumorigenic human lung bronchial epithelial cells to a tumorigenic state, enabling them to grow as poorly differentiated squamous tumors in immunocompromised mice.

**Figure 8:**
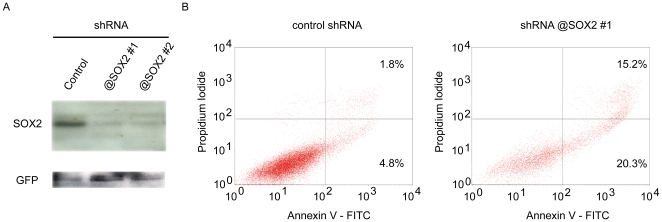
Consequences of shRNA-mediated knockdown of SOX2. **A.** Western blot analysis of cells transfected with SOX2-specific shRNA-expressing plasmids. Two shRNAs were found to very efficiently knockdown SOX2 protein expression in 293T cells over-expressing SOX2 (see Materials and Methods). **B**. FACS analysis of AnnexinV and Propidium Iodide stained BEAS-2B cells. Control and SOX2 shRNA-transduced cells were stained to determine the proportions of cell death by FACS analysis. Proportions of early apoptotic (AnnexinV+/PI-) and late apoptotic/necrotic (AnnexinV+/PI+) cells are strikingly increased in SOX2 knockdown cells.

Finally, we assessed the consequences of SOX2 knockdown in lung squamous cell lines using two independent shRNAs that very efficiently knockdown SOX2 protein expression (Figure 8 panel A). Upon lentiviral-mediated transduction of the three lung squamous cell lines with these two SOX2-targeting shRNAs, we observed massive death within 10–20 days (depending on the cell line), whereas control-transduced cells re-grew normally. Three independent experiments (with the two SOX2 targeting shRNAs and the three cell lines, BEAS-2B, NCI-H226 and Calu-1) failed to establish stably transduced cells, showing that SOX2 expression is critical for the survival of these cell lines. Consequently, we explored the cell death mechanism at an early time point after positive selection of transduced cells by FACS analysis using Annexin V and Propidium Iodide (PI) staining. An increase of early apoptotic (AnnexinV+/PI-) and late apoptotic/necrotic (AnnexinV+/PI+) cell populations were observed in the SOX2 knockdown cells, as was shown for the BEAS-2B cells (Figure 8 panel B). Altogether, these results unambiguously demonstrate that SOX2 expression is essential to lung squamous cell line survival.

## Discussion

Using array-CGH, we characterized chromosome 3 aberrations in a series of 26 lung SCCs. The short arm of chromosome 3 displays several discontinuous sites of recurrent losses resulting in combined allelic losses, which may affect different TSGs involved in lung tumorigenesis [35]. The *OGG1* and *RAD18* loci were the most frequently lost (60% of cases) in our study, and these genes are good TSG candidates given their DNA repair-related functions. On the long arm, the whole 3q26-qter region commonly (around 60%) experiences low-level gains. Therein, the *PIK3CA* and *RFC4* loci are gained in a wide majority (≥80%) of advanced lung SCCs, as previously reported [5], [6]. PIK3CA is a well-established oncogene [36] and RFC4 is recurrently over-expressed in a broad range of solid tumor types [26].

High-level gene amplifications have been reported for several 3q26-qter region loci [6], [7]. Incidentally, we found sporadic amplifications of the *EIF4G1* and *EIF5A2* loci (one to two tumors each). More consistently, we uncovered high-level amplifications of the *SOX2* region (2 Mb around this gene) as the most frequent chromosome 3 amplification site in three independent cohorts (>130 lung SCCs in total), with about 20% of tumors carrying 3q26.33 amplifications. This site is also among the most frequently amplified in the whole genome of lung SCCs. This finding is in line with the amplification rate of canonical oncogenes, such as *EGFR*, *ERBB2, C-MYC*, *N-MYC*, *CCND1, MDM2*, and *RAS*, which generally range from 10 to 30% in different tumor types [2]. Therefore, the high occurrence of 3q26.33 amplifications in lung SCCs likely reflects a critical cellular selective advantage conferred by the activated oncogene(s) in this region. In addition, the very high frequency of copy number increases of the locus (60 to 80% of tumors with gains) strongly suggests that these aberrations may be constitutive or at least a very common theme of lung SCC carcinogenesis and possibly also cervical cancer.

Gene amplification is a known mechanism for oncogene activation and is implicated in tumor progression by the step-wise selection of tumor cells with increasing malignant capabilities [2], [3]. Within an amplicon, the expression of several genes is usually affected by gene dosage. By-stander genes can be up-regulated due to their position, but do not necessarily bring any functional advantage to the tumor cells. By contrast, driver genes are over-expressed as a result of copy number increases and functionally participate in the malignant phenotype. In this report, most of the nine genes mapping in the 3q26.33 core amplicon were found to be over-expressed due to copy number increases, with the exception of *LAMP3*. Among them, *SOX2*, *DCUN1D1*, *FXR1* and *ATP11B* are also over-expressed in lung SCC when compared to normal lung in published studies in which genomic copy numbers were unknown. These genes may be commonly involved in lung carcinogenesis and knowledge of their normal function may indicate their relevance to carcinogenesis. In that respect, the oncogenic potential *DCUN1D1* was proposed to act through the Gli1/Hedgehog signaling pathway [8], which is active in less than 10% of lung SCCs [37]. Sarkaria et al. demonstrated that *DCUN1D1* over-expression transforms NIH-3T3 fibroblasts and renders them tumorigenic *in vivo*. More recently, this group showed that DCUN1D1 participates in cullin neddylation [38] and induces NIH-3T3 fibroblast invasion *in vitro* by activating MMP-2 expression [39]. Its yeast and nematode homologues play a role in mitotic spindle regulation [40] and act as scaffolding E3 ubiquitin ligases critical for cullin neddylation [41]. *DCUN1D1* over-expression could thus have pleiotropic effects and may be a sound candidate driver oncogene of 3q26.3 amplifications in lung SCCs.

Here, we describe high *SOX2* over-expression as a direct consequence of 3q26.33 copy number increases and found that SOX2 is activated in a wide majority of lung SCCs (67%). Accordingly, we demonstrated that over-expression of SOX2 in lung epithelial cells promoted cell migration and anchorage-independent growth *in vitro*. We showed that SOX2 over-expression can be transforming and lead to the growth of poorly differentiated basaloid variants of squamous tumors in immunocompromised mice [33], [34]. In addition, SOX2 expression was found to be critical for survival of lung cell lines, as indicated by SOX2 silencing RNA interference experiments. These findings unambiguously indicate the oncogenic properties of SOX2 and designate it as a driver gene. Our results, together with previous characterizations of *DCUN1D1* function, suggest that 3q26.3 may be a new example of a multi-oncogenic locus [9]. *DCUN1D1* and *SOX2* may be co-driver genes of 3q26.3 amplification that synergistically modulate different molecular traits and result in a more aggressive tumor phenotype. Further investigations are required to elucidate their cooperation and respective involvement in carcinogenesis. Such experiments should involve concomitant functional analyses of the modulated phenotypical traits *in vivo*. We propose a model where SOX2 over-expression confers higher tumorigenicity and contributes to loss of differentiation by lung squamous epithelial cells and where DCUN1D1 increases invasive capabilities through MMP2 [39] along with other effects [38], [40], [41].

We demonstrate striking and systematic up-regulation of *SOX2* and *SOX2OT* among tumors with either 3q26.3 amplifications or gains. Concomitant over-expression of both genes suggests that they may be co-regulated. *SOX2* is contained in an intron with the SOX2 Overlapping Transcript encoding gene. Both genes are transcribed in the same orientation. SOX2OT is thus not an antisense RNA, but a large non-coding RNA with an elusive role. Deciphering its function remains of interest, especially in light of the growing body of evidence for the importance of non-coding RNAs in physiopathological situations, such as cancer [42]. *SOX2* is a functionally well-characterized transcription factor and an outstanding oncogene candidate. Our finding of frequent and intense SOX2 nuclear staining in lung SCC is consistent with its role in gene expression regulation.

In normal fetal chick and murine lungs, Sox2 is expressed in the primary bronchus epithelium, but not in distal bronchiolar buds [16], [43], [44]. In the adult murine lung, Sox2 is expressed by ciliated epithelial cells and induced in bronchiolar epithelial ciliated, squamous and cuboidal cells upon injury [45]. Over-expression of Sox2 in transgenic mouse lung epithelium leads to an increase in committed precursor-like cells, notably basal cells, and reveals its role in branching morphogenesis as well as in the control of lung epithelial cell differentiation [16]. Apparently contradictory with our results, it has also been reported that SOX2 over-expressing lungs are reduced in size, which may result from a slight but not statistically significant reduction in proliferation [16]. However, given the cellular diversity of the lung tract (and localization differences of these cell types between mouse and human) and the absence of definite identification of the cell of origin of lung SCC, extrapolation of SOX2 cellular consequences in the context of lung cancer from mouse normal lung development data is difficult.

Here we show that Sox2 is expressed in normal human lungs in an overall similar pattern to that for the mouse. It will be of further interest to determine the extent to which Sox2 participates in human lung epithelial stem and progenitor cell homeostases, as in the mouse [16], [45].

In tumors, SOX2 is up-regulated in early pancreatic cancer lesions [46], where it is nuclear in basal cells or in the vicinity of necrotic areas and is activated in a majority of advanced tumors [47]. SOX2 is associated with an immature phenotype in CNS teratomas and glioblastomas [48]. In breast cancer, it has been proposed that 3q gains activate *SOX2*, thereby driving a basal cell-like phenotype [49]. In the MCF-7 breast cancer cell line, SOX2 over-expression stimulates anchorage-independent growth and tumor growth, while its knockdown produces converse effects [50]. These data are in accordance with our results obtained in lung squamous cell lines. Chen et al, [50] also report that the SOX2-positive action on breast cancer cell proliferation and tumorigenesis is mediated by synergistic activation of *CCND1* expression with β-catenin. Finally, SOX2-activated target genes (that have been defined as such in hESCs) are up-regulated in basal-like breast tumors and in poorly differentiated grade 3 breast tumors [27].

In lung SCC, we have shown here that SOX2 over-expression may contribute to direct cell cycle regulation and proliferation by affecting key regulators of the cell cycle, with exclusion of *CCND1*. Key transcription factors controlling wide transcriptional programs, such as SOX2, rely on regulatory networks to ensure tissue- and time-specific modulation of their transcriptional programs [51]. The global readout is highly dependent on the availability of the partners in a given cell type, especially for SOX proteins [52]. Such cell type specificity in cofactors may account for some of the differences observed downstream of SOX2 activation in breast cancer and lung SCC. The 16 cancer-relevant gene list that we generated, which includes genes that are likely targets of *SOX2* and altered in lung SCC, is a good starting point to evaluate the molecular mechanisms downstream of SOX2. We have shown that SOX2 over-expression is capable of transforming non-tumorigenic lung squamous BEAS-2B cells, enabling them to form slowly growing tumors *in vivo*. A possible explanation for this relatively mild transforming power is that additional hits are required downstream or in cooperation with SOX2 activation and that a specific genetic makeup is required in the tumor to fully allow for SOX2 oncogenic action. It will therefore be of further interest to determine which alterations are capable of cooperating with SOX2 activation in lung SCC. In addition, our results reveal activation of embryonic and neural SC molecular phenotypes in lung SCC and support a major contribution of SOX2 thereto *in vivo*. Finally, the hESC consensus and hESC-like gene modules, which are activated in poorly-differentiated and aggressive epithelial cancers [27], [28], are commonly activated in lung SCCs as well. SOX2 may be directly involved in this process, as we have shown. These results led us to speculate that SOX2 induces a poorly differentiated state that may favor cellular plasticity and help cells to cope with the changing environment during tumor progression. Interestingly, Sox2 is expressed by cancer stem cells (CSCs, Cancer Stem Cells, cancer cells with tumor-initiating properties) derived from pediatric brain cancers [53], and SOX2 knockdown arrests proliferation and results in a loss of tumorigenicity of glioblastoma CSCs [54]. Such CSCs have since been identified in various solid tumors, including squamous tumors [55]–[57]. Given the role of SOX2 in the control of normal embryonic and neural SCs homeostases and our identification of *SOX2* as a novel oncogene in lung SCC, it will be of interest to dissect its involvement in cancer stem cells (CSCs) isolated from squamous tumors.

Our study shows that Sox2 is an oncogene in lung SCC and a strong candidate for driving the recurrent 3q26.33 amplifications in these tumors. Sox2 provides a major contribution to transcriptome deregulation and is thus very likely to exert pleiotropic effects on lung SCC carcinogenesis. During the manuscript submission process, a report on the role of SOX2 as an oncogene in lung SCC was published [58], providing corroborative evidence for our conclusions. Further *in vivo* studies will be critical to comprehensively dissect SOX2-specific effects. We speculate that SOX2 might be involved in several steps during lung SCC progression. First, SOX2 is capable of transforming and conferring tumor-initiating properties to human lung squamous cells, as we demonstrated. Second, SOX2 contributes to establishment of a stem cell-like poorly-differentiated phenotype in advanced lung SCC, as is also suggested for SOX2 in basal-like breast cancer [27], [49] and other tumor types [48]. Further investigations are required to assess whether Sox2 plays a role in the homeostases of normal bronchiolar epithelial stem cells and of lung squamous cancer stem cells. This work requires isolation of the normal lung and cancer stem cells and the identification of the cells-of-origin of lung SCC. Altogether, SOX2 could participate in tumor progression at several steps during carcinogenesis by promoting both tumor cell de-differentiation and maintenance of a stem/progenitor cell-like phenotype.

## Materials and Methods

### Ethics Statements

Human tumor samples came from a Grenoble biobank approved by the French Research Ministerium with patient informed consent (written approval N° AC-2007-23). Data were analyzed anonymously. Mouse experiments were performed in accordance with Swiss guidelines and approved by the Veterinarian Office of Vaud, Switzerland.

### Samples, Cell Lines and Transductions

Patients were untreated prior to tumor excision. DNA was extracted using the phenol/chloroform method, and RNA was extracted using Trizol (Invitrogen). BEAS-2B (an immortalized but non-tumorigenic cell line in nude mice, CRL-9609), NCI-H226 (CRL-5826), and Calu-1 (HTB-54) cell lines originally established from SSC tumors (and tumorigenic in nude mice) were obtained from ATCC (http://www.atcc.org/). BEAS-2B was grown in RPMI 1640 without Hepes (Gibco) and 10% fetal calf serum and gentamicine. NCI-H226 was grown in RPMI 1640 without Hepes (Gibco), 10 mM Hepes, 2.5 g/L glucose, 1 mM Sodium Pyruvate, 10% fetal calf serum and gentamicine. Calu-1 was grown in MEM alpha medium (Gibco), 10% fetal calf serum and gentamicine. These cell lines were transduced using pRRL third-generation lentiviral vectors [59] for GFP and luciferase (control) or GFP and Sox2 expression. Transduced cells were sorted by FACS (based on GFP expression) and re-cultured for expansion.

### Array-Comparative Genomic Hybridization

Two high-resolution chromosome 3 arrays, prepared as previously described [60], were used. The “chromosome 3” array served to analyze the initial 26 tumor series. The “contig” array (an updated version that provides a tiling coverage of the 3q26.33 locus) was used to reanalyze tumor DNAs with 3q26.3 amplifications found in the first screen. Data are available in GEO (GSE15080). IntegraChip™ BAC pangenomic arrays (IntegraGen) were also used to analyze two tumors (#15 and 35). Publicly available array-CGH data were downloaded from GEO. Details on data sources and other supplementary methods are given in Text S1.

### RT-PCR and Immunohistochemistry

RT-qPCR and immunohistochemistry were performed as described [61]–[63].

### Genome-Wide Gene Expression and Bioinformatic Analyses

We analyzed transcriptome data by comparing lung SCC and normal lung tissue data [20], [21]. BEAS-2B (Sox2 vs. control) cells were profiled following standard protocols (HG U133+2.0 arrays, IGBMC Affymetrix core facility). Details are given in Text S1 and Genelist S1.

### shRNAs

A control scramble shRNA and two different shRNAs targeting SOX2 in pLKO.1-puro were used (OpenBiosystems; shRNA#1, clone ID TRCN0000085750; shRNA#2, clone ID TRCN0000085749). SOX2-targeting shRNAs were validated upon transient co-transfections of a SOX2 (and GFP) expression construct and either a control shRNA or a SOX2-targeting shRNA in 293T cells. Upon lentiviral-mediated transductions of lung squamous cells using these constructs, transduced cells were selected by adding 1 µg/ml puromycin (Sigma) to the media at 48 h post-transduction.

### FACS Analysis of Annexin V and PI Stained Cells

Control- and SOX2-shRNA-transduced cells were stained using AnnexinV-FITC (BD Biosciences) and Propidium Iodide (Sigma) 5 days after puromycin selection and were analyzed on a FACS Calibur.

### Tumor Growth In Vivo

In vivo tumor growth assays followed institutional and national regulations. Female NMRI nude mice (6–8 weeks old, Elevage Janvier, France) were submitted to subcutaneous injections of 2.5×10^6^ cells in 100 µl PBS. Each animal received both control- (left flank) and Sox2-transduced (right flank) cells. In one experiment, four animals were tested with each cell line (BEAS-2B and NCI-H226).

### Accession Numbers

The MIAME-compliant microarray data are available in GEO (SuperSeries record GSE15080).

## Supporting Information

Text S1 Additional Materials and Methods information.
(0.09 MB PDF)

Figure S1 Array-CGH analyses of lung and uterine cervix SCCs. A. Whole genome array-CGH profiles for lung tumors #15 (left panel) and 35 (right panel). For both tumors, the 3q26.33 amplification (green arrow) represents the highest copy number increase detected over the entire genome. Chromosome numbers are indicated above the graph, and the different chromosomes are separated by the vertical lines. B. Amplification frequencies of chromosome 3 loci in two independent cohorts of uterine cervix SCCs. In the two cohorts, the maxima of amplification are observed for the 3q26.33 locus (green arrow). C. Individual Chromosome 3 array-CGH profiles obtained for lung tumors #15 and 35 reanalyzed with the 3q26.3 tiling array. The green dashed rectangles indicate the regions presented in panel D. D. Individual array-CGH profiles for tumors #15 and 35 with the 3q26.3 tiling array: insets of the chromosome 3 interval from 170 to 190 Mb.
(1.03 MB TIF)

Figure S2 GSEA-based enrichment analyses of lung SCC dataset-2. A. Enrichments of human ESC-like molecular phenotypes among the genes deregulated in lung SCC dataset-2. The human ESC consensus gene module (FDR <10-2; left panel) and the human ESC-like gene module (FDR <10-2, right panel) are significantly enriched among the genes over-expressed in lung SCCs. B. Enrichments of *SOX2* targets in human ESCs among the genes deregulated in lung SCC dataset-2. Genes that are known, direct *SOX2*-activated targets in human ESCs are significantly enriched among genes over-expressed in lung SCCs (FDR <0.02, left panel). Genes that are known *SOX2*-repressed targets in human ESCs are significantly enriched among genes down-regulated in lung SCCs (FDR <0.05, right panel).
(1.01 MB TIF)

Figure S3 Characterization of the *SOX2*-squamous signature. A. Venn diagram representing the overlap between the *SOX2* squamous signature and the *SOX2* targets in human ESCs. Gene numbers are indicated in the corresponding section. The p-value indicates the significance of overlap (hypergeometric distribution). B. Significantly Enriched Gene Ontology Biological processes in the *SOX2*-squamous signature. C. Significantly Enriched Gene Ontology Cellular compartments in the *SOX2*-squamous signature. Panels B and C: All represented categories are significantly enriched (FDR<10-2 and p<10-5). D. Enrichments of the human ESC-like gene module upon *SOX2* over-expression in BEAS-2B cells. A significant portion (292 genes, 23%) of the human ESC-like gene module is activated upon *SOX2* over-expression in BEAS-2B cells (FDR  = 0.12).
(0.67 MB TIF)

Figure S4 Western blot analysis of *SOX2* expression in transduced lung squamous cell lines. BEAS-2B, NCI-H226, and Calu-1 cells were transduced with a *SOX2* lentiviral vector (or control). For each cell line, protein extracts were submitted to western blot analysis after normalization of protein loading. Endogenous *SOX2* is visible as a thin band in the three cell lines with increased expression in NCI-H226, which contains a gain of this genomic regions by array-CGH. This form probably corresponds to a posttranslational modified protein, such as that described previously [66]. A similar pattern of migration was observed in *SOX2* over-expressing cells; see figure 2B in [67]. Phosphorylation and sumoylation have also been reported for *SOX2*. On the contrary, cell lines transduced with a *SOX2* plasmid produced a large amount of unmodified *SOX2*.
(0.06 MB PDF)

Figure S5 Effects of *SOX2* over-expression in BEAS-2B cells on anchorage-independent growth. A. Colony number quantification. *SOX2* over-expression leads to a significant increase in BEAS-2B colony number. Of note, these colonies become are largely visible macroscopically upon *SOX2* over-expression. B. Colony size quantification. *SOX2* over-expression leads to a significant increase in colony size. Representative images of colonies from BEAS-2B control and *SOX2* cell lines are presented.
(3.30 MB TIF)

Table S1 The 50 most over-expressed genes in lung SCC versus normal comparison (dataset 1). Gene Symbol, Gene description, Ratio (log2 value) lung SCC/normal.
(0.04 MB XLS)

Table S2 *SOX2* immunostainings individual results for 51 lung SCC. Individual nuclear and cytoplasmic staining scores obtained for the 51 primary tumors. The 5 tumours found with high level amplifications in the initial arrayCGH screen of 26 tumours are indicated in bold.
(0.04 MB XLS)

Table S3 Known *SOX2* target genes significantly correlated to *SOX2* expression in human lung SCC. Union of the 71 *SOX2* activated target genes significantly correlated to *SOX2* expression and the 26 *SOX2* repressed genes significantly anti-correlated to its expression in lung SCC (97 genes in total).
(0.04 MB XLS)

Table S4 Union of the *SOX2*-squamous and the lung SCC signatures. Union of the *SOX2*-squamous and either of the lung SCC signatures (142 genes in total). Expression ratios (log2 values) are indicated.
(0.04 MB XLS)

Genelist S1 Supplementary genelists xls file, several sheets.
(0.42 MB XLS)
