# Supplementary material for: SOX2 Is an Oncogene Activated by Recurrent 3q26.3 Amplifications in Human Lung Squamous Cell Carcinomas
Source: PLoS One. 2010 Jan 29;5(1):e8960. doi: 10.1371/journal.pone.0008960 (PMC2813300; doi:10.1371/journal.pone.0008960)
Supplement: Text S1 — Additional Materials and Methods information. (0.09 MB PDF) [file pone.0008960.s001.pdf]

Supplementary Information file

"*SOX2* is an Oncogene Activated by Recurrent 3q26.3 Amplifications in Lung Squamous Cell Carcinomas"

Hussenet et al.

A. Microarrays : data sources, availability and analyses

A.1 Array-CGH

A.1.1 Microarrays production for array-CGH

A.1.2 Array-CGH data availability and analyses

A.1.2.1 Chromosome 3 array-CGH screening in 26 lung SCC

A.1.2.2 Whole genome array-CGH studies of independent SCC cohorts

A. 2 Expression microarrays and data mining

A. 2.1 Oncomine analyses of 3q26.3 genes expression in lung SCC

A.2.2 Analysis of genome-wide gene expression profiling data

A. 2.2.1 Data sources

A. 2.2.2 Data analyses : definition of signatures and genesets

A. 2.2.3. Integrative analyses and functional annotations

B. RT-quantitative PCR and primers

C. Immunohistochemistry

D. Soft Agar, wound healing and invasion assays

E. Weblinks

F. References

A. Microarrays : data sources, availability and analyses

All mapping values are from UCSC genome browser [1,2] July 2003 version corresponding to NCBI build 34 of the human genome reference sequence.

MIAME-compliant microarray data we produced is available from GEO using the SuperSeries accession record GSE15080 (encompassing Series GSE14859, GSE5079 and GSE14883).

A.1. Array-CGH

A.1.1 Microarrays production for array-CGH

The two "chromosome 3" and "3q26.3 contig" arrays were prepared as previously described [3] and descriptions of the two arrays are available in GEO under accession numbers GPL8186 and GPL8187. IntegraChip^{TM} BAC pangenomic arrays (IntegraGen, Evry, France) were also used to analyze two tumours (# 15 and 35; fig. S1A).

A.1.2 Array-CGH data availability and analyses

A.1.2.1 Chromosome 3 arrayCGH screening in 26 lung SCC

ArrayCGH data we produced is available in GEO for both the initial screen of chromosome 3 aberrations in the 26 lung SCC (fig. 1A-B; GEO Series record GSE14859) and the precise 3q26.33 amplicon mapping for the 5 tumours using the 3q26.33 tiling array (fig. 1E and S1C-D; GSE15079).

Data were analyzed as described previously [3] : for each array, we excluded from analyses the clones that displayed signal intensities for control (normal) DNA hybridization lower than twice the local background values for more than one of the three replicates present on the array. After subtraction of local background, we calculated the ratio of Cy5 (tumor) and Cy3 (normal) intensities and values were considered as valid only if the coefficient of variation (standard deviation divided by the arithmetic mean) of the replicates was less than 20%. To determine the normal genomic ratio, we calculated the median of the ratios obtained for the subtelomeric clones (representing all sub-telomeric regions of the genome). Chromosome 3 clones individual ratios were then normalized to 1 using this median. We used the values of 2, 1.25, and 0.75 as thresholds for amplifications, gains, and losses, respectively.

Chromosome 3 regions associated with Copy Number Variants (as listed in the CNV database, cf weblinks) were excluded from analyses.

A.1.2.2 Whole genome array-CGH studies of independent SCC cohorts

Data corresponding to the profiling of whole genome aberrations in 34 lung SCC (fig. 1C-D, GSE12280, [4]) and 10 and 47 uterine cervix SCC (fig. S1C, GSE6473, [5]; fig. S1B GSE11573, [26]) were all downloaded from GEO. The log2 normalized available array-CGH data were used. Clones for which a ratio tumor/normal was measured in less than half of the samples only were excluded. For a given clone, high-level amplifications were considered when a log2 ratio tumor/normal > 1.5 (lung SCC) or > 1 (uterine cervix SCC) was obtained. Amplification frequencies were measured by dividing the number of amplifications

occurrences by the number of measures and expressing it as a percentage. BAC clones that were found with multiple mappings on the human genome were excluded from analyses.

A. 2 Expression microarrays and data mining

A. 2.1 Oncomine analyses of 3q26.3 genes expression in lung SCC (fig. 2A-B)

The Oncomine database [6] was used to monitor gene expression of genes located in the 3q26.3 core amplicon, in available independent transcriptome profiling of

a) 13 lung SCC compared to 5 normal lung tissue using cDNAs microarrays [7] (fig. 2A). *SOX2* is represented by the IMAGE clone # 786674 in this microarray dataset and was found not to be deregulated in the tumors. However, this clone is chimeric and thus we excluded it from further analyses. The corresponding cDNA sequences deposited in Genbank indeed reveal that the 3'end contains Sox2 sequence (AA451892, BX118929), whereas the 5'end is composed of another gene (AA452080), thus not allowing to reliably measure Sox2 expression.

b) 21 human lung SCC compared to 17 normal lung tissue transcriptome profiling using Affymetrix HG-U95Av2 arrays [8] (fig. 2B).
The *DCUN1D1* gene could not be analyzed since not represented on this array.

A.2.2 Analysis of genome-wide gene expression profiling data

A. 2.2.1 Data sources

Raw affymetrix CEL files from 21 human lung SCC and 17 normal lung tissue transcriptome profiling [8] were downloaded from the author's website (cf weblinks). This dataset is referred as to the **lung SCC dataset-1**.

Normalized log2 data corresponding to the profiling of 13 lung SCC and 6 normal lung tissue using cDNA microarrays [7] were downloaded from GEO (GSE3398). For our analyses, we discarded the 1 normal fetal lung sample and conserved the 5 adult normal lung samples. This dataset is referred as to the **lung SCC dataset-2**.

Transduced BEAS-2B -SOX2 and -control cells were profiled using Affymetrix HG-U133 Plus 2.0. Data are available in GEO (GSE14883).

The na27 affymetrix annotations release (November 2008) was used for arrays annotations.

A. 2.2.2 Data analyses : definition of signatures and genesets

All therafter defined signatures and genesets are available in the corresponding supplementary xls file accompanying this article and avaible online from the editor's website.

For Affymetrix arrays, RMA normalization and differential expression analyses were performed using the "Remote Analysis Computation for gene Expression data" tool (RACE) [9]. Deregulated genes were selected based on expression ratio test/reference >log2 (1.5) or

<log2 (3/2) and $p < 0.05$ for statistical significance. Raw data from Bhattacharjee et al. [8] were also independently re-analyzed using Genepattern [10] (fig. 2C).
For cDNA microarrays [7], normalized and log2 transformed data were used to select genes based on expression ratio test/reference >log2 (3) or <log2 (1/3) and $p < 0.05$ (Student t-Test). These selection steps lead to the establishment of :

- the **lung SCC signature-1:** 1203 annotated genes (1394 probesets, ~ 11 % of total probesets) differentially expressed in lung SCC versus normal lung (dataset-1) [8].

- the **lung SCC signature-2:** 975 annotated genes (1012 cDNAs, ~ 4.4 % of total cDNAs) differentially expressed in lung SCC versus normal lung (dataset-2) [7].

- the **SOX2-squamous signature :** 922 annotated genes (1378 probesets, ~ 2.5% of total) differentially expressed in BEAS-2B SOX2 versus control.

We also derived or used genes modules/genesets from previously published works and that represent experimentally defined but curated and validated genesets:

a) **SOX2 target genes in human ESCs genesets**

We used the list of genes bound by SOX2 in human ESCs from ChIP experiments [11] and retrieved their expression level in human ESCs versus differentiated cells from a meta-analysis [12]. Genes that were found bound by SOX2 and upregulated in hESCs versus differentiated cells were considered as SOX2 activated direct target genes in hESCs (242 genes in total). Genes that were found bound and downregulated in hESCs versus differentiated cells were considered as SOX2 repressed direct target genes (103 in total).

b) c-MYC targets geneset
We used three lists of consensus c-Myc target genes in various cellular systems [13-15]. Corresponding genesets were downloaded from MSigDB (names of the genesets: FERNANDEZ_MYC_TARGETS, MYC_TARGETS, MYC_ONCOGENIC_SIGNATURE). We fused these three lists of consensus c-Myc targets, discarded redundant ones and obtained a 415 individual c-Myc targets genelist used in our analyses.

c) Embryonic Stem Cell-like gene modules

- human ESC consensus gene module
It results from the meta-analysis of 20 different human ESCs profiling studies [12] and consists in 379 genes that are found overexpressed in hESCs in at least 5 of these studies. This geneset was originally defined as the “ES EXP1 set” and used to demonstrate the presence of an embryonic stem cell-like molecular phenotype in poorly differentiated breast cancer and its association to poor prognosis [16]. We renamed it as the human ESC consensus gene module for clarity purposes.

- human ESC-like gene module
It was previously defined [17] and consists in the genes overlapping in gene expression profiling experiments of human ECs (Embryonic Carcinoma) and human ESCs and comprises 1247 individual human genes. It is found activated in several poorly differentiated human primary tumors, and its activation is correlated to poor patient prognosis in breast and lung adenocarcinoma [17].

A. 2.2.3. Integrative analyses and functional annotations

We used :

i) Database for Annotation, Visualization and Integrated Discovery (DAVID) [18], to retrieve significantly enriched ($p < 10^{-5}$, $FDR < 10^{-2}$) Gene Ontologies for the SOX2-squamous signature (figure S3B-C).

ii) Molecular Signatures Database (MSigDB, cf the weblinks section) [19] to compute overlaps between the signatures we defined and genesets that represent gene expression modules/signatures grouping functionally related genes based on their genomic co-localization or co-expression in previously published profiling studies (fig. 4A). The online available query tool was used to compute significant enrichments in the lung SCC signatures using all available molecular signatures available in the database (n=5452 in total). Statistical significance of enrichments is assessed by hypergeometric distribution.

iii) Gene Sets Enrichment Analysis (GSEA) [19, 20] to compute the significant overlaps between defined genesets and genome-wide expression datasets (fig. 4B,4D,4E, 5A, S2, and S3D).
Datasets were collapsed to Gene Symbols for analysis. Only enrichments with an $FDR \leq 0.25$ were considered [19]. Analyses results with $0.05 < FDR \leq 0.25$ were considered as relatively enriched whereas results with $FDR < 0.05$ were considered to represent statistically significant enrichments.

Enrichments of the hESC consensus and hESC-like gene modules in the lung SCC datasets -1 and -2 (fig. 4B and S2A, respectively): comparisons with the lung SCC datasets were done by using phenotype permutations (n=1000) and the Signal2Noise metric to rank genes in the datasets. Enrichments of SOX2 target genes in the lung SCC datasets -1 and -2 (fig. 4D and S2B, respectively) were analyzed similarly.

Enrichments of the hESC consensus and hESC-like gene modules in the lung SCC datasets -1 using SOX2 expression to define phenotypes within the dataset (fig. 4E): phenotypes were created by GSEA using the SOX2 affymterix probeset 33108_i_at and genes were ranked in the dataset according to their correlation/anti-correlation to SOX2 expression. Phenotype permutations (n=1000) and the Pearson metric were used to rank genes in the datasets.

iv) Genmapp [21] to visualize cell cycle regulators significantly enriched among the genes that are known SOX2 targets and correlating to its expression in primary lung SCC (fig. 5C). The pathway mapp is as available in Genmapp and was originally derived from the KEGG pathway database (cf weblinks).

B. RT-quantitativePCR and primers

Primers were designed using Primer3 software [22]. Total RNA were submitted to DNase I digestion (1h at 25°C) and reverse-transcription reactions performed on 500ng RNA (Superscript II RT Kit, Invitrogen). RPLP0 mRNA was used as internal control for quantitativePCR (Lightcycler, Roche; as in [23,24]). Experiments (from RNA extractions to qPCR reactions) were performed three times independently and results averaged.

Sequences of primers used are :

GENE	FORWARD Primer sequence (5' -> 3')	REVERSE Primer sequence (5' -> 3')
FXR1	ATCAGCGTGACAGCAGGAGA	TGAGATTCGCTGGCATCAGT
DNAJC19	GGCCTGGCAACTTGGATACT	TGAGGCTGCAATGAACTGTG
SOX2	GACCAGCTCGCAGACCTACA	CCGGGGAGATACATGCTGAT
SOX2OT	GCTGGGAAGGACAGTTCGAG	AGCCACTGAAAGGCAAGGTC
ATP11B	AGTCCAACCCACATCAGCAG	CCTTACCCCAAAGCCACAAA
DUNC1D1	GCATGGCCTGTTCTTATTGATG	AATGTTTAGAGCGGCCCAGA
B3GNT5	GTGGTGCCCCTCCCATTAG	CAGAGGCCCATGAACACATC
LAMP3	CAAACAGCGGCCACAGTAAA	TATGAGCTGGTGGGGTGATG
MCCC1	TCCAGGATGCGGTTTTCTTT	TCAGGCACTGGTCTGATTGG
RPLP0	GAAGGCTGTGGTGCTGATGG	CCGGATATGAGGCAGCAGTT

C._Immunohistochemistry

SOX2 immunostainings were performed using a three step immunoperoxidase method on a series of 51 advanced lung SCC (26 initially analyzed by array-CGH and 25 tumors in addition). After antigen retrieval (15 minutes microwave in sodium citrate buffer pH6), the primary antibody (AB5603, Chemicon International) was applied at 1/1000 dilution overnight at 4°C. Donkey anti-rabbit (1/1250) biotinylated antibody and streptavidin-biotin-peroxidase complex were then used, followed by diaminobenzidine for development. For each tumor, SOX2 IHC quantification was performed using the signal score method, evaluated for both cytoplasmic and nuclear staining in lung SCC cells. IHC for SOX2, Keratins 5/6 and Ki67 were performed similarly on paraffin embedded subcutaneous tumors. Negative controls were performed by omitting the primary antibodies.

Scoring the results of SOX2 IHC in the 51 primary tumors was performed as previously [25]. For a given tumor: staining strength (from 0 to 3; 0 = absent, 1 = low, 2 = intermediate, 3 = strong) is multiplied by the percentage of positive tumor cell nuclei or cytoplasm (ranging from 0 to 100). Both a “cytoplasmic signal score” and a “nuclear signal score” were calculated for each case. The final scores range from 0 to 300 (0 = no expression in any cell; 300 = strong expression in 100% of cells).

D._Soft-agar, invasion and wound healing assays

5. 10^3 BEAS-2B -control or - SOX2 cells were plated in normal medium containing 0.3% soft agar on top of a 0.6% agar layer and plates incubated at 37°C for 3 weeks. Colonies were stained using crystal violet. Both macroscopic and microscopic images were acquired using a camera and phase contrast on an inverted microscope, respectively. Image quantifications of microscopic images (colony number and size) were made using the ImageJ software (NIH). The experiment was replicated three times independently and measures subsequently averaged.
For invasion assays, Growth factor reduced Matrigel Chambers were used (BD Biosciences) : 2.10^5 cells were loaded in medium containing 0.1% serum in the upper insert and allowed to invade towards medium 10% serum. Experiments were performed in triplicate and subsequently averaged.
Wound healing assays were performed by plating 2.5-5 10^5 cells per well in 6-well plates and allowed to form a confluent monolayer for 48h. Scratches were made using a P200 tip, and an image acquired. An other image was acquired after 16h (NCI-H226) or 24h (BEAS-2B and Calu-1). Image J was used to measure wound size initially and at the end of the experiment to quantify wound closure which is expressed as the percentage of closure of the initial wound. For one condtion, 3 wounds were created per experiment. Experiment were reproduced three times independently and measures subsequently averaged.

E. Weblinks

Bhattacharjee et al., (2001) Raw data
http://www.broad.mit.edu/mpr/lung/

CNV database
http://projects.tcag.ca/variation/

DAVID
http://david.abcc.ncifcrf.gov/

Gene Expression Omnibus
http://www.ncbi.nlm.nih.gov/geo/

GenePattern
http://www.broad.mit.edu/cancer/software/genepattern/

Gene Sets Enrichment Analysis
http://www.broad.mit.edu/gsea/

Genmapp
http://www.genmapp.org/

KEGG pathway database
http://www.genome.ad.jp/kegg/pathway.html

Molecular Signatures Database

http://www.broad.mit.edu/gsea/msigdb/index.jsp

Oncomine
http://www.oncomine.org/main/index.jsp

Primer3
http://frodo.wi.mit.edu/cgi-bin/primer3/primer3_www.cgi

RACE
http://race.unil.ch/

UCSC Genome Browser
http://genome.ucsc.edu

F. References

1: Kent WJ, Sugnet CW, Furey TS, Roskin, KM, Pringle TH et al. (2002) The Human Genome Browser at UCSC. Genome Res. 12: 996-1006.
2: Karolchik D., Baertsch R., Diekhans M, Furey TS, Hinrichs A et al. (2003) The UCSC Genome Browser Database. Nucl. Acids Res. 31: 51-54.
3: Hussenet T, Mallem N, Redon R, Jost B, Aurias A et al. (2006) Overlapping 3q28 amplifications in the COMA cell line and undifferentiated primary sarcoma. Cancer Genet Cytogenet 169: 102-113.
4: Boelens MC, Kok K, van der Vlies P, van der Vries G et al. (2009) Genomic aberrations in squamous cell lung carcinoma related to lymph node or distant metastasis. Lung Cancer. 2009 Mar 24. [Epub ahead of print]
5: Wilting SM, de Wilde J, Meijer CJ, Berkhof J et al. (2008) Integrated genomic and transcriptional profiling identifies chromosomal loci with altered gene expression in cervical cancer. Genes Chromosomes Cancer 47: 890-905.
6: Rhodes DR, Kalyana-Sundaram S, Mahavisno V, Varambally R, Yu J et al. (2007) Oncomine 3.0: genes, pathways, and networks in a collection of 18,000 cancer gene expression profiles. Neoplasia 9: 166-180.
7: Garber ME, Troyanskaya OG, Schluens K, Petersen S, Thaesler Z et al.. (2001) Diversity of gene expression in adenocarcinoma of the lung. Proc Natl Acad Sci U S A 98: 13784-13789.
8: Bhattacharjee A, Richards WG, Staunton J, Li C, Monti S, Vasa P, et al. (2001) Classification of human lung carcinomas by mRNA expression profiling reveals distinct adenocarcinoma subclasses. Proc Natl Acad Sci U S A 98: 13790-13795.
9: Psarros M, Heber S, Sick M, Thoppae G, Harshman K et al. (2005) RACE: Remote Analysis Computation for gene Expression data. Nucleic Acids Res. 1: W638-643.
10: Reich M, Liefeld T, Gould J, Lerner J, Tamayo P et al. (2006) GenePattern 2.0. Nat Genet. 38: 500-501.
11: Boyer LA, Lee TI, Cole MF, Johnstone SE, Levine SS et al. (2005) Core transcriptional regulatory circuitry in human embryonic stem cells. Cell 122: 947-956.
12: Assou S, Le Carrour T, Tondeur S, Ström S, Gabelle A et al (2007) A meta-analysis of human embryonic stem cells transcriptome integrated into a web-based expression atlas. Stem Cells 25: 961-973.
13: Fernandez PC, Frank SR, Wang L, Schroeder M, Liu S, Greene J, Cocito A, Amati B. (2003). Genomic targets of the human c-Myc protein. Genes Dev. 17, 1115-11129.
14: Zeller KI, Jegga AG, Aronow BJ, O'Donnell KA, Dang CV (2003) An integrated database of genes responsive to the Myc oncogenic transcription factor: identification of direct genomic targets. Genome Biol. 4: R69.
15: Bild AH, Yao G, Chang JT, Wang Q, Potti A et al. (2006) Oncogenic pathway signatures in human cancers as a guide to targeted therapies. Nature 439: 353-357.
16: Ben-Porath I, Thomson MW, Carey VJ, Ge R, Bell GW et al. (2008) An embryonic stem cell-like gene expression signature in poorly differentiated aggressive human tumors. Nat Genet. 40: 499-507.
17: Wong DJ, Liu H, Ridky TW, Cassarino D, Segal E et al. (2008) Module map of stem cell genes guides creation of epithelial cancer stem cells. Cell Stem Cell 2: 333-344.
18: Dennis G Jr, Sherman BT, Hosack DA, Yang J, Gao W et al. (2003) DAVID: Database for Annotation, Visualization, and Integrated Discovery. Genome Biol. 4, P3.
19: Subramanian A, Tamayo P, Mootha VK, Mukherjee S, Ebert BL et al. (2005) Gene set enrichment analysis: a knowledge-based approach for interpreting genome-wide expression profiles. Proc Natl Acad Sci U S A. 102: 15545-15550.
20: Mootha VK, Lindgren CM, Eriksson KF, Subramanian A, Sihag S et al. (2003) PGC-1alpha-responsive genes involved in oxidative phosphorylation are coordinately downregulated in human diabetes. Nat Genet. 34: 267-273.
21: Salomonis N, Hanspers K, Zambon AC, Vranizan K, Lawlor SC et al. (2007) GenMAPP 2: new features and resources for pathway analysis. BMC Bioinformatics 8: 217.
22: Rozen S, Skaletsky H. (2000) Primer3 on the WWW for general users and for biologist programmers. In: Krawetz S, Misener S (eds). Bioinformatics Methods and Protocols: Methods in Molecular Biology. Humana Press, Totowa, NJ, pp 365-386.
23: Redon R, Hussenet T, Bour G, Caulee K, Jost B et al. (2002) Amplicon mapping and transcriptional analysis pinpoint cyclin L as a candidate oncogene in head and neck cancer. Cancer Res 62: 6211-6217.
24: Muller D, Millon R, Theobald S, Hussenet T, Wasylyk B et al. (2006) Cyclin L1 (CCNL1) gene alterations in human head and neck squamous cell carcinoma. Br J Cancer 94: 1041-1044.
25: Lantuejoul S, Soria JC, Morat L, Lorimier P, Moro-Sibilot D et al. (2005). Telomere shortening and telomerase reverse transcriptase expression in preinvasive bronchial lesions. Clin Cancer Res. 11: 2074-2082.
26: Wilting SM, Steenbergen RD, Tijssen M, van Wieringen WN et al. (2009) Chromosomal signatures of a subset of high-grade premalignant cervical lesions closely resemble invasive carcinomas. Cancer Res 69: 647-55.
