# Supplementary figures and images for: SOX2 Is an Oncogene Activated by Recurrent 3q26.3 Amplifications in Human Lung Squamous Cell Carcinomas
Source: PLoS One. 2010 Jan 29;5(1):e8960. doi: 10.1371/journal.pone.0008960 (PMC2813300; doi:10.1371/journal.pone.0008960)

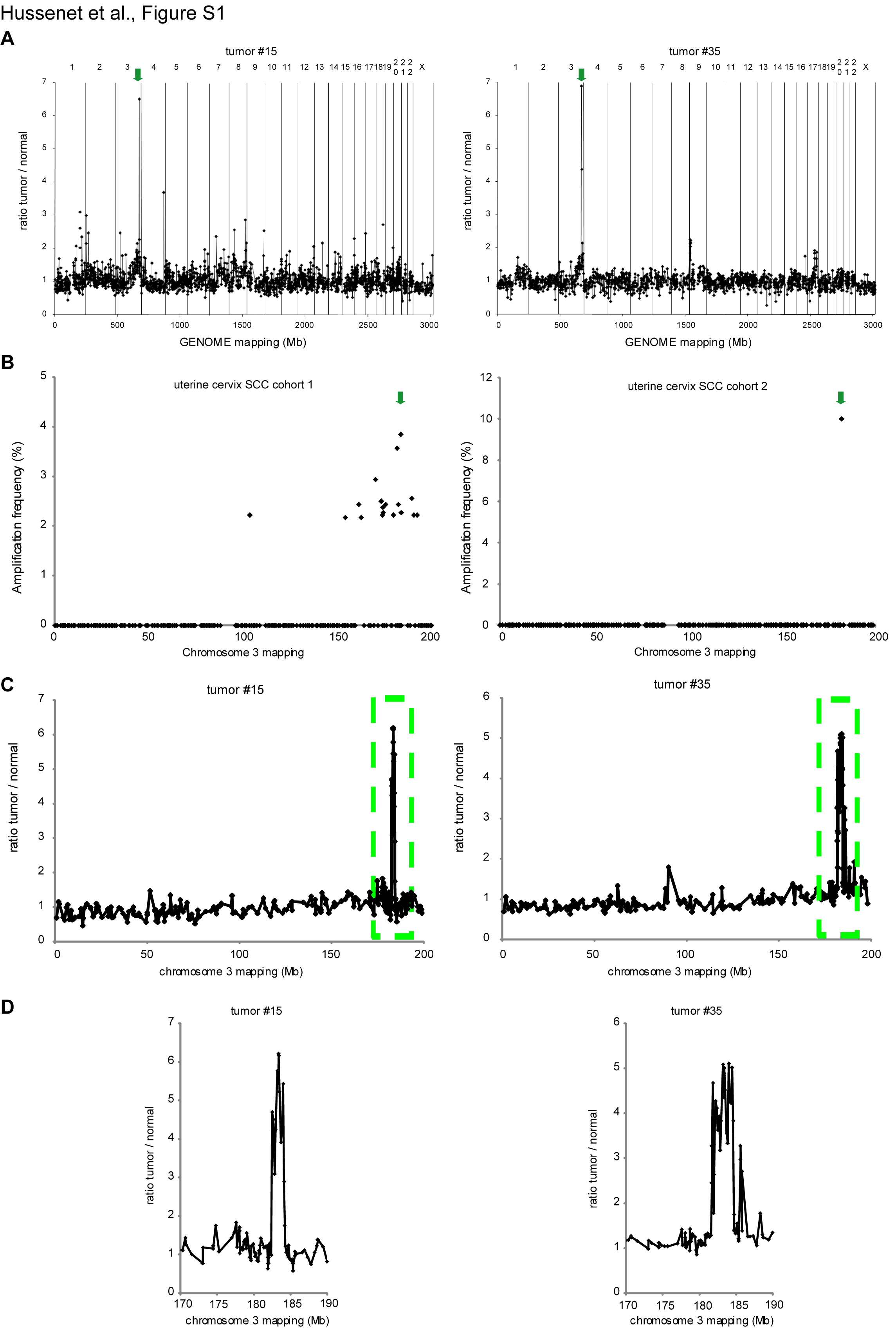

Supplement: Figure S1 — Array-CGH analyses of lung and uterine cervix SCCs. A. Whole genome array-CGH profiles for lung tumors #15 (left panel) and 35 (right panel). For both tumors, the 3q26.33 amplification (green arrow) represents the highest copy number increase detected over the entire genome. Chromosome numbers are indicated above the graph, and the different chromosomes are separated by the vertical lines. B. Amplification frequencies of chromosome 3 loci in two independent cohorts of uterine cervix SCCs. In the two cohorts, the maxima of amplification are observed for the 3q26.33 locus (green arrow). C. Individual Chromosome 3 array-CGH profiles obtained for lung tumors #15 and 35 reanalyzed with the 3q26.3 tiling array. The green dashed rectangles indicate the regions presented in panel D. D. Individual array-CGH profiles for tumors #15 and 35 with the 3q26.3 tiling array: insets of the chromosome 3 interval from 170 to 190 Mb. (1.03 MB TIF) [file pone.0008960.s002.tif]

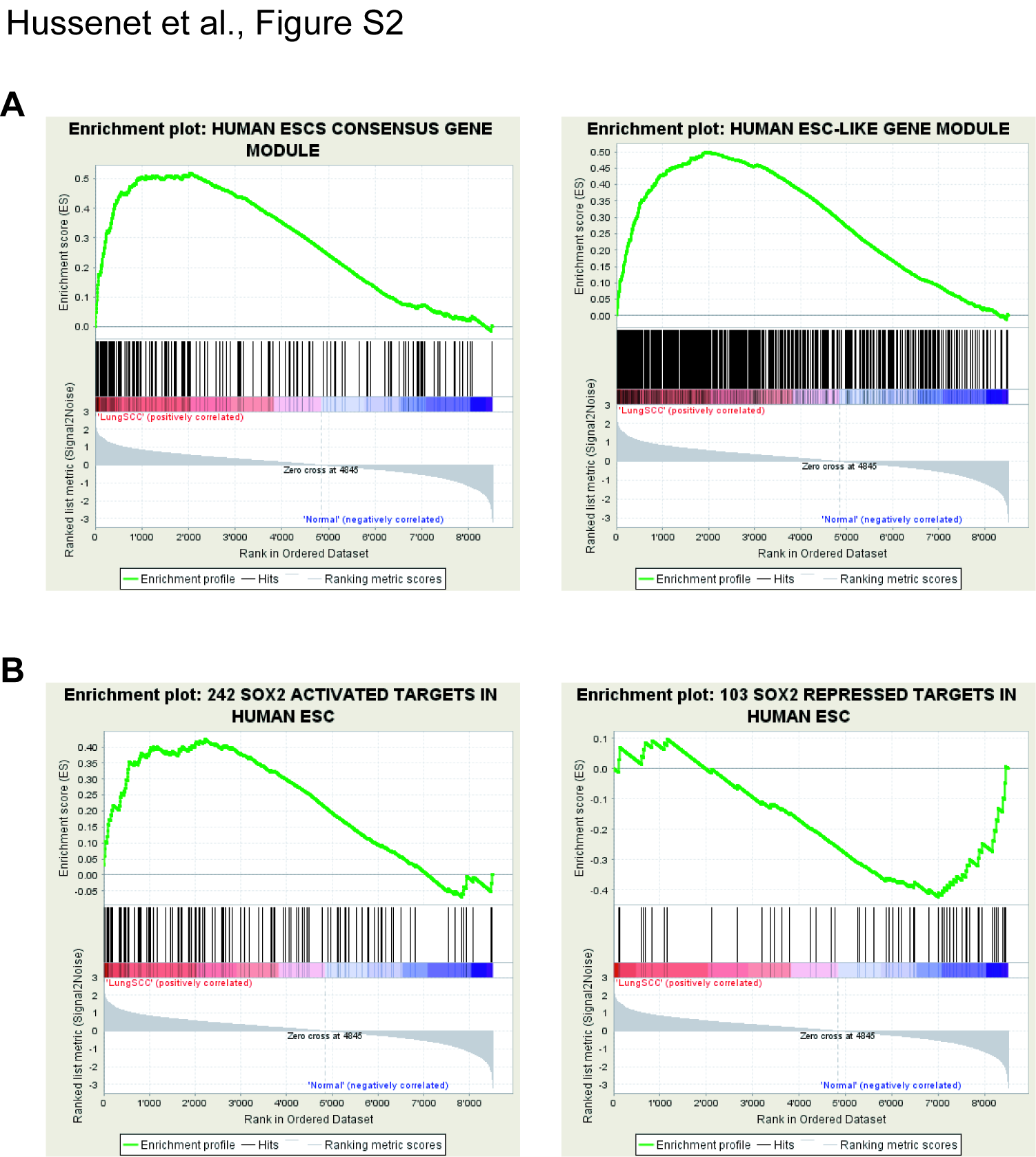

Supplement: Figure S2 — GSEA-based enrichment analyses of lung SCC dataset-2. A. Enrichments of human ESC-like molecular phenotypes among the genes deregulated in lung SCC dataset-2. The human ESC consensus gene module (FDR <10-2; left panel) and the human ESC-like gene module (FDR <10-2, right panel) are significantly enriched among the genes over-expressed in lung SCCs. B. Enrichments of SOX2 targets in human ESCs among the genes deregulated in lung SCC dataset-2. Genes that are known, direct SOX2-activated targets in human ESCs are significantly enriched among genes over-expressed in lung SCCs (FDR <0.02, left panel). Genes that are known SOX2-repressed targets in human ESCs are significantly enriched among genes down-regulated in lung SCCs (FDR <0.05, right panel). (1.01 MB TIF) [file pone.0008960.s003.tif]

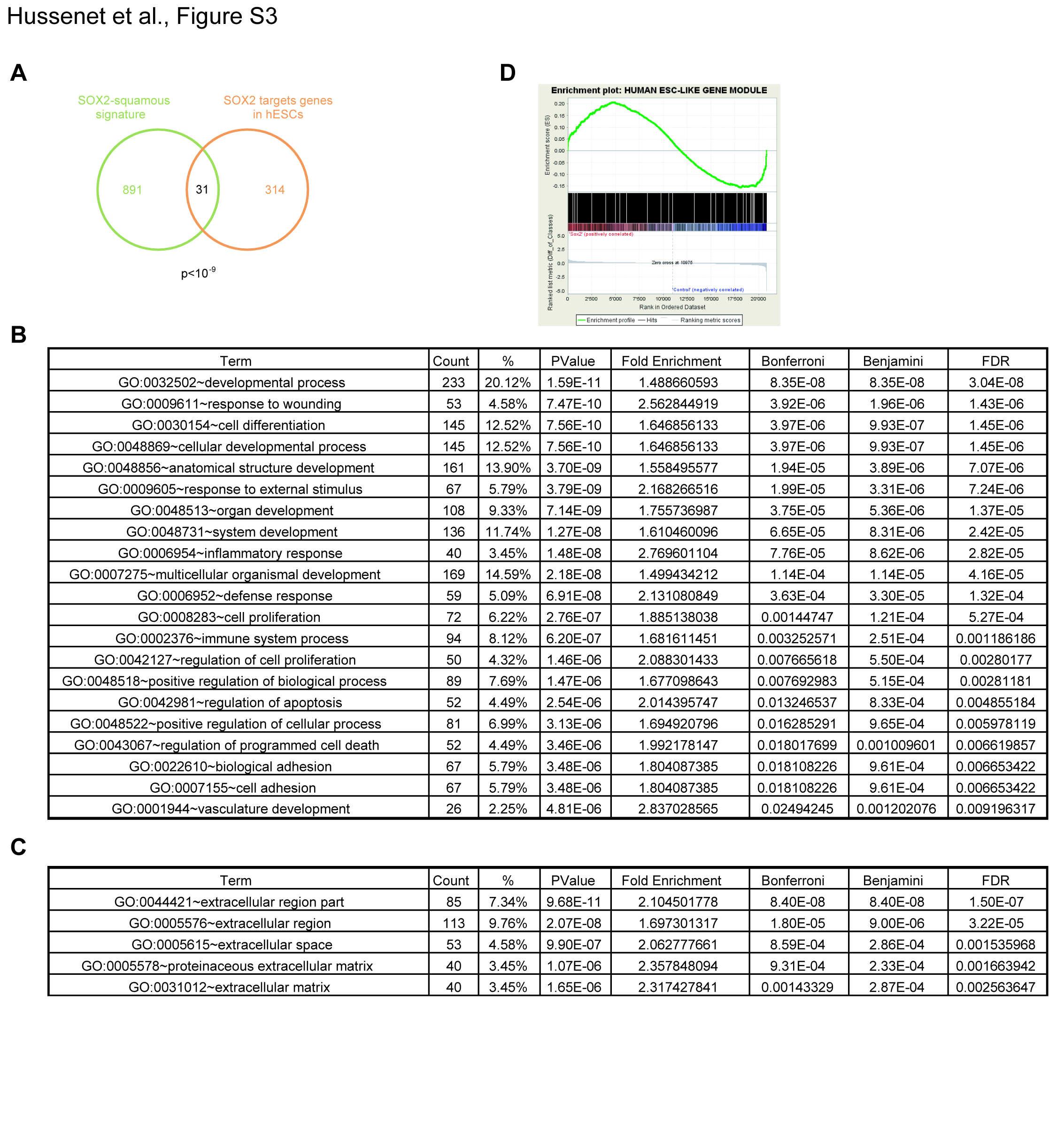

Supplement: Figure S3 — Characterization of the SOX2-squamous signature. A. Venn diagram representing the overlap between the SOX2 squamous signature and the SOX2 targets in human ESCs. Gene numbers are indicated in the corresponding section. The p-value indicates the significance of overlap (hypergeometric distribution). B. Significantly Enriched Gene Ontology Biological processes in the SOX2-squamous signature. C. Significantly Enriched Gene Ontology Cellular compartments in the SOX2-squamous signature. Panels B and C: All represented categories are significantly enriched (FDR<10-2 and p<10-5). D. Enrichments of the human ESC-like gene module upon SOX2 over-expression in BEAS-2B cells. A significant portion (292 genes, 23%) of the human ESC-like gene module is activated upon SOX2 over-expression in BEAS-2B cells (FDR = 0.12). (0.67 MB TIF) [file pone.0008960.s004.tif]

# Hussenet et al., Figure S4

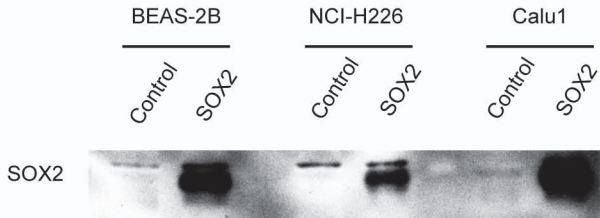

Supplement: Figure S4 — Western blot analysis of SOX2 expression in transduced lung squamous cell lines. BEAS-2B, NCI-H226, and Calu-1 cells were transduced with a SOX2 lentiviral vector (or control). For each cell line, protein extracts were submitted to western blot analysis after normalization of protein loading. Endogenous SOX2 is visible as a thin band in the three cell lines with increased expression in NCI-H226, which contains a gain of this genomic regions by array-CGH. This form probably corresponds to a posttranslational modified protein, such as that described previously [66]. A similar pattern of migration was observed in SOX2 over-expressing cells; see figure 2B in [67]. Phosphorylation and sumoylation have also been reported for SOX2. On the contrary, cell lines transduced with a SOX2 plasmid produced a large amount of unmodified SOX2. (0.06 MB PDF) [file pone.0008960.s005.pdf]

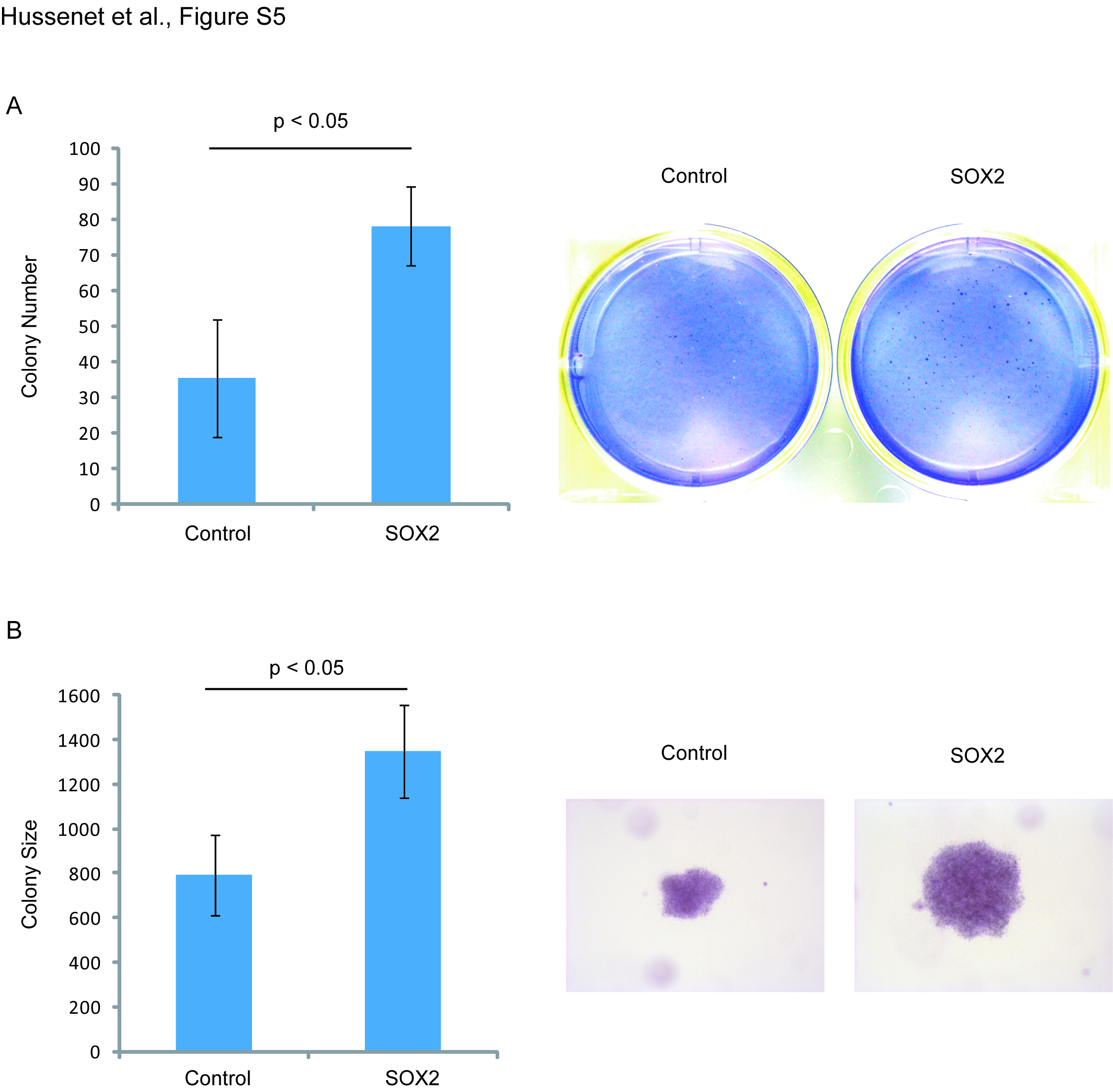

Supplement: Figure S5 — Effects of SOX2 over-expression in BEAS-2B cells on anchorage-independent growth. A. Colony number quantification. SOX2 over-expression leads to a significant increase in BEAS-2B colony number. Of note, these colonies become are largely visible macroscopically upon SOX2 over-expression. B. Colony size quantification. SOX2 over-expression leads to a significant increase in colony size. Representative images of colonies from BEAS-2B control and SOX2 cell lines are presented. (3.30 MB TIF) [file pone.0008960.s006.tif]
